# Changes in protein fluxes in skeletal muscle during sequential stages of muscle regeneration after acute injury in male mice

**DOI:** 10.1038/s41598-024-62115-x

**Published:** 2024-06-07

**Authors:** Alec Bizieff, Maggie Cheng, Kelvin Chang, Hussein Mohammed, Naveed Ziari, Edna Nyangau, Mark Fitch, Marc K. Hellerstein

**Affiliations:** Division of Metabolic Biology, Department of Nutritional Sciences & Toxicology, University of California-Berkeley, Berkeley, CA USA

**Keywords:** Muscle damage, Muscle injury, In-vivo regeneration, Flux proteomics, Stable isotope labeling, Mass spectrometry, Mass spectrometry, Muscle stem cells, Regeneration

## Abstract

Changes in protein turnover play an important role in dynamic physiological processes, including skeletal muscle regeneration, which occurs as an essential part of tissue repair after injury. The inability of muscle tissue to recapitulate this regenerative process can lead to the manifestation of clinical symptoms in various musculoskeletal diseases, including muscular dystrophies and pathological atrophy. Here, we employed a workflow that couples deuterated water (^2^H_2_O) administration with mass spectrometry (MS) to systematically measure in-vivo protein turnover rates across the muscle proteome in 8-week-old male C57BL6/J mice. We compared the turnover kinetics of over 100 proteins in response to cardiotoxin (CTX) induced muscle damage and regeneration at unique sequential stages along the regeneration timeline. This analysis is compared to gene expression data from mRNA-sequencing (mRNA-seq) from the same tissue. The data reveals quantitative protein flux signatures in response to necrotic damage, in addition to sequential differences in cell proliferation, energy metabolism, and contractile gene expression. Interestingly, the mRNA changes correlated poorly with changes in protein synthesis rates, consistent with post-transcriptional control mechanisms. In summary, the experiments described here reveal the signatures and timing of protein flux changes during skeletal muscle regeneration, as well as the inability of mRNA expression measurements to reveal changes in directly measured protein turnover rates. The results of this work described here provide a better understanding of the muscle regeneration process and could help to identify potential biomarkers or therapeutic targets.

## Introduction

A unique feature of skeletal muscle tissue is that it can recapitulate certain elements of embryonic myogenesis upon injury, by replacing damaged muscle fibers with newly formed ones. This is an important process that allows tissue to recover from and adapt to repeated bouts of stress, like those incurred during physical activity or exercise^1^. The regenerative capacity of skeletal muscle comes from a small portion of peripherally located progenitor stem cells, known as muscle satellite cells (MuSCs)^2–4^. An impairment in this repair process may contribute to signs and symptoms of musculoskeletal diseases such as muscular dystrophy and aging-related atrophy^5,6^. Understanding skeletal muscle regeneration can lead to rational countermeasures against the impairment of regenerative capacity.

Traditional abundance-based measurements can establish changes in protein expression. However, the transition from MuSCs to mature myofibers is a dynamic process that involves synchronized, time-dependent changes in protein fluxes. Traditional abundance-based measurements cannot capture the dimension of protein fluxes^7^. Skeletal muscle regeneration has been previously divided into distinct stages that follow a sequential order: initial tissue damage, inflammation-mediated immune response, activation/proliferation of MuSCs, differentiation of MuSCs to myofibers, and maturation of new myofibers into existing tissue^8^. To undergo complete skeletal muscle regeneration, the rates of protein synthesis and/or degradation must be altered in the transition from one stage to the next. A kinetic signature in the form of newly synthesized proteins may therefore be an identifiable characteristic of each stage.

Here, we employed a workflow or measurement technique to measure the kinetic signatures of each stage of muscle regeneration by in-vivo labeling of proteins with deuterium (^2^H) from deuterated “heavy” water (^2^H_2_O). Changes in abundances and patterns of mass isotopomers in skeletal muscle proteins were quantified by high-performance liquid chromatography (HPLC) single and tandem mass spectrometry (MS/MS) and used to measure changes in protein flux rates, based on combinatorial probabilities (mass isotopomer distribution analysis [MIDA]) as previously described^9–11^. We asked whether the use of this flux proteomics approach would identify global, group, and individual protein kinetic changes at each stage. This information on protein turnover rates was then compared to gene level expression values during in-vivo muscle regeneration. The overarching goal of this work was to expand the understanding of the in-vivo muscle regeneration process by elucidating protein flux signatures at various stages and comparing them to gene expression values. The information from this work can then be used to establish a reference for the identification of potential biomarkers or therapeutic targets in the various stages of muscle regeneration.

## Methods

### Male mouse experiments

#### Ethical approval

For this study, 20 male 8-week-old C57/BL6 mice (weighing 20–30 g) were purchased from Jackson Laboratory (Bar Harbor, ME) and housed at UC Berkeley’s Northwest Animal Facility. All animal housing and experiments in this study were approved by and carried out according to the Animal Care and Use Committee (ACUC) standards at UC Berkeley, Institutional Ethics Committee approval number: AUP-2014-12-7005-3. All groups of mice had unrestricted access to an ad libitum standard chow diet and 8% Deuterium drinking water (^2^H_2_O). ^2^H_2_O was provided to continually label the mice after a bolus intraperitoneal injection of 35 ml/gram bodyweight 99.1% ^2^H_2_O/0.9% saline solution at the beginning of the incubation to allow for stable enrichment levels during the entire labeling time course. Exclusion criteria were established before the experiments began and stated that if a mouse appeared to have issues accessing food or water due to the experimental model at any time during incubation, then they were removed from the study and sacrificed. No mice were excluded and sacrificed early due to any adverse reactions to these experiments. The authors confirm that all methods reported in this paper are reported in accordance with the ARRIVE 2.0 guidelines^12^.

#### CTX time course

The mice were randomly assigned to a group (n = 5 per group) that reflected the stage of muscle regeneration being measured (Proliferation Stage, Differentiation Stage, Maturation Stage, or Uninjured Control). No confounders were controlled for group randomization and researchers were blinded to experimental and control groups. CTX-treated groups received a dose of 50 µL 0.1 mg/mL CTX in sterile PBS/0.2% Meloxicam in the form of an intramuscular injection in the Tibialis Anterior (TA) muscle to one limb while the TA from the other limb was not injected. The day of CTX injection is called Day 0, and subsequent days allow for the toxin-induced muscle regeneration to occur. One group of mice did not receive CTX injection to serve as uninjured controls but did receive 3 days of stable isotope labeling alongside experimental groups. At specific time points along the muscle regeneration timeline after initial CTX injection pertaining to unique stages (Proliferation Stage: 4 days after injection, Differentiation Stage: 7 days after injection, Maturation Stage: 14 days after injection), animals were anesthetized by inhalation of a steady stream of 2.5% isoflurane in oxygen, sacrificed by cervical dislocation under anesthesia, and tissues were taken for analysis. To avoid bias from multiple analyses, all animals were entered into the study at the same time and age and sacrificed on Day 14 of the study. Muscle histology and protein expression via immunofluorescence was utilized to confirm each stage of muscle regeneration. After sacrifice, target tissues (multiple muscles including TA, blood [serum], kidneys, liver) were collected along with urine, as previously described^13^, for the following procedures.

#### Immunofluorescence

Briefly, tissues were harvested and snap-frozen in OCT. 10 µm sections were taken from the muscle’s mid-belly with a cryostat and placed on positively charged frosted slides for imaging. Slides with tissue sections were washed 3 times with PBS-T (0.1% Triton-X 100) for 5 min each and then air dried for 15 min at room temperature. Slides were blocked in buffer (10% Normal Donkey Serum (NDS) + 1% Bovine Serum Albumin (BSA) in PBS-T) for 1 h at room temperature. Primary antibodies (Embryonic Myosin Heavy Chain: #F1.652 clone, Developmental Studies Hybridoma Bank, University of Iowa, deposited by Blau, HM) 1:10. β-Laminin: #AF-3837, R&D Systems, 1:20) were added in the same buffer used to block the slides and allowed to incubate in a dark, humid container overnight at 4 °C. The next day, samples were washed 3 times with PBS-T for 5 min each. Secondary antibodies (Donkey anti Goat, Alexa Fluor 488, Abcam, #AB150129, 1:500. Donkey anti Mouse, Alexa Fluor 647, Abcam, #AB150107, 1:500) were added in the same buffer used to block the samples for 1 h at room temperature, in the dark. Samples were washed 3 more times with PBS-T for 5 min each, dried, and mounted with 3 drops of Fluoromount (Sigma Aldrich #F4680) mounting media and cover slips. A Zeiss Axioscope fluorescence microscope was used for fluorescent imaging.

#### Tissue histology

Hematoxylin and eosin staining was performed on 10 µm sections of regenerating muscle on positively charged glass slides, as previously described^14^ (DMD_M.1.2.007). Images were collected with a Zeiss Plan-Apochromat 20x/0.8NA (WD = 0.55 mm) M27 Biomarker Technology Core microscope. Imaging was conducted in a Zeiss Axio Scan.Z1 whole slide scanner objective lens in the brightfield mode with Hitachi HV-F202 camera.

#### Visual quantification of images

Quantification of images for visible muscle damage, tissue regeneration, and protein expression and protein level analysis via immunofluorescence was carried out using ImageJ software (https://imagej.nih.gov/). In brief, the entire surface area of the tissue was quantified with ImageJ and compared to either the entire surface area of the damaged/regenerating tissue or the number of myofibers expressing the protein of interest. Visible areas of freeze-fracture and tissue overlap in the images were excluded from surface area analysis. Also, protein levels of individual myofibers were measured and averaged by image staining intensity with the software. The individual doing the analysis was blinded by a Python script that generated random image codes for file names. Images were later decoded after visual quantification for analysis of each stage. Significance for all quantification analyses of tissue images was determined by One-Way ANOVA.

#### Body water determination

Mouse blood was distilled overnight upside down on a bead bath at 85 °C to evaporate out body water. Deuterium present in the body water was exchanged onto acetone, and deuterium enrichment in the body water was measured via gas chromatography mass-spectrometry (GC–MS), as previously described^15^.

### RNA-sequencing

#### RNA bulk isolation

Whole RNA transcripts were isolated from thawed aliquots of single muscle fibers with attached fiber-associated cells, such as satellite cells, according to manufacturer's instructions (Qiagen RNA Extraction Mini Kit, #74104), and RNA concentrations were obtained using a Nanodrop.

#### Sample collection and preparation

From the RNA sample to the final data, each step, including sample test, library preparation, and sequencing, influences the quality of the data, and data quality directly impacts the analysis results. To guarantee the reliability of the data, quality control (QC) is performed at each step of the procedure.

#### RNA quantification and qualification

RNA degradation and contamination was monitored on 1% agarose gels. RNA purity was checked using the NanoPhotometer^®^ spectrophotometer (IMPLEN, CA, USA). RNA integrity and quantitation were assessed using the RNA Nano 6000 Assay Kit of the Bioanalyzer 2100 system (Agilent Technologies, CA, USA).

#### Library preparation for transcriptome sequencing

A total amount of 1 μg RNA per sample was used as input material for the RNA sample preparations. Sequencing libraries were generated using NEBNext^®^ UltraTM RNA Library Prep Kit for Illumina^®^ (NEB, USA) following manufacturer’s recommendations and index codes were added to attribute sequences to each sample. Briefly, mRNA was purified from total RNA using poly-T oligo-attached magnetic beads. Fragmentation was carried out using divalent cations under elevated temperature in NEBNext First Strand Synthesis Reaction Buffer (5×). First strand cDNA was synthesized using random hexamer primer and M-MuLV Reverse Transcriptase (RNase H-). Second strand cDNA synthesis was subsequently performed using DNA Polymerase I and RNase H. Remaining overhangs were converted into blunt ends via exonuclease/polymerase activities. After adenylation of 3ʹ ends of DNA fragments, NEBNext Adaptor with hairpin loop structure were ligated to prepare for hybridization. To select cDNA fragments of preferentially 150–200 bp in length, the library fragments were purified with AMPure XP system (Beckman Coulter, Beverly, USA). Then 3 μl USER Enzyme (NEB, USA) was used with size-selected, adaptor-ligated cDNA at 37 °C for 15 min followed by 5 min at 95 °C before PCR. Then PCR was performed with Phusion High-Fidelity DNA polymerase, Universal PCR primers and Index (X) Primer. At last, PCR products were purified (AMPure XP system) and library quality was assessed on the Agilent Bioanalyzer 2100 system.

#### Clustering and sequencing

The clustering of the index-coded samples was performed on an Illumina Novaseq sequencer according to the manufacturer’s instructions. After cluster generation, the libraries were sequenced on the same machine and paired-end reads were generated.

### Sample digestion for proteomics

#### Muscle fiber isolation and CD45+ cell depletion via MACS

Tissues were snap-frozen after harvest in 0.5 mL of 10% DMSO in FBS and stored at – 80 °C until processing. Samples were thawed, buffer was removed, and individual muscle fibers were physically separated from whole muscle tissue, as previously described^16^. Single muscle fibers with attached fiber-associated cells were then depleted of infiltrating CD45+ cells by undergoing magnetic assisted cell sorting (MACS) mediated selective depletion, according to manufacturer's instructions (Miltenyi Biotec #130-052-301), before being processed for in-solution digestion.

#### Myofiber SDS solubilization + in-solution digestion of tissue

After MACS-mediated CD45+ cell depletion, a 150 µL aliquot of single muscle fibers with attached fiber-associated cells was brought up to 500 µL in 0.1% SDS solution and allowed to disassociate on a vortexer overnight at medium speed. The next day, samples were pulled off the vortexer, mixed with 7:1 v/v 100% ethanol:sample, vortexed, and placed at − 20 °C overnight to precipitate out proteins. The next day, samples were centrifuged 16,000*g* for 30 min at 4 °C to pellet out all protein. Supernatant was removed and protein pellet was resuspended in 100 µL 8 M Urea in 50 mM Ammonium Bicarbonate (pH 8.1) with agitation on vortexer at medium speed for 30 min at room temperature. A small portion of each sample was diluted, and protein concentration was then determined by the Pierce BCA protein assay kit (Thermo Fisher #23225) with BSA as standards. Up to 100 µg of protein was taken and volume adjusted up to 100 µL in 8 M Urea in 50 mM Ammonium Bicarbonate (pH 8.1). Tris-(2-carboxyethyl)-phosphine (TCEP) was added to make a final concentration of 10 mM and samples were agitated on a vortexer for 20 min at room temperature. Iodoacetamide (IAA) was added to make a final concentration of 20 mM, samples were vortexed briefly, and incubated at room temperature in the dark for 30 min. TCEP was added to make a final concentration of 4 mM to quench excess IAA and samples were diluted in 50 mM Ammonium Bicarbonate (pH 8.1) so that the final concentration of Urea was < 1 M. Proteomics-Grade Trypsin was added at a ratio of 1:50 trypsin to protein (Sigma Aldrich, #T6567). Samples were incubated at 37 °C overnight. The next day, formic acid was added at 5% of the final volume. Samples underwent Solid-Phase Extraction using Agilent C18 clean up columns (#A57203) to remove digested peptides from the digestion buffer. Peptides were eluted using 30% acetonitrile and speedvac’d until dry and re-suspended in 25 µL of 0.1% formic acid/3% acetonitrile/96.9% LC–MS grade water and transferred to LC–MS vials to be analyzed via LC–MS.

#### Mitochondria isolation for proteomics

After MACS-mediated CD45+ cell type depletion, a 150 µL aliquot of single muscle fibers with attached fiber-associated cells was used to isolate mitochondrial proteins according to manufacturer’s instructions (Thermo Fisher #89801). Protein pellets were resuspended in 50 mM Ammonium Bicarbonate (pH 8.1) and protein concentration was then determined by the Pierce BCA protein assay kit with BSA as standards. Up to 100 µg of protein was taken and volume adjusted to 100 µL in 50 mM Ammonium Bicarbonate (pH 8.1). TCEP was added to make a final concentration of 10 mM and samples were agitated on a vortexer for 20 min at room temperature. IAA was added to make a final concentration of 20 mM, samples were vortexed briefly, and incubated at room temperature in the dark for 30 min. TCEP was added to make a final concentration of 4 mM to quench excess IAA and samples were diluted in 50 mM Ammonium Bicarbonate (pH 8.1) up to 400 µL. Proteomics-Grade Trypsin was added at a ratio of 1:50 trypsin to protein. Samples were incubated at 37 °C overnight. The next day, formic acid was added at 5% of the final volume. Samples were centrifuged at 10,000*g* for 10 min at room temperature, and the supernatant was collected. Supernatant was speedvac’d until dry and re-suspended in 50 µL of 0.1% formic acid/3% acetonitrile/96.9% LC–MS grade water and transferred to LC–MS vials to be analyzed via LC–MS.

#### DNA incorporation of ^2^H

After MACS mediated CD45+ cell type depletion, a 150 µL aliquot of single muscle fibers with attached fiber-associated cells was used to extract DNA. DNA was derivatized and analyzed for deuterium enrichment in deoxyribose via gas chromatography mass spectrometry, as previously described^17,18^.

#### Liquid chromatography–mass spectrometry (LC–MS) analysis

Trypsin-digested peptides were analyzed on a 6550 quadropole time of flight (Q-ToF) mass spectrometer equipped with Chip Cube nano ESI source (Agilent Technologies). High performance liquid chromatography (HPLC) separated the peptides using capillary and nano binary flow. Mobile phases were 95% acetonitrile/0.1% formic acid in LC–MS grade water. Peptides were eluted at 350 nl/min flow rate with an 18-min LC gradient. Each sample was analyzed once for protein/peptide identification in data-dependent MS/MS mode and once for peptide isotope analysis in MS mode. Acquired MS/MS spectra were extracted and searched using Spectrum Mill Proteomics Workbench software (Agilent Technologies) and a mouse protein database (http://www.uniprot.org). Search results were validated with a global false discovery rate of 1%. A filtered list of peptides was collapsed into a nonredundant peptide formula database containing peptide elemental composition, mass, and retention time. This was used to extract mass isotope abundances (M_0_–M_3_) of each peptide from MS-only acquisition files with Mass Hunter Qualitative Analysis software (Agilent Technologies). Mass isotopomer distribution analysis (MIDA) was used to calculate peptide elemental composition and curve-fit parameters for predicting peptide isotope enrichment based on precursor body water enrichment (p) and the number (n) of amino acid C-H positions per peptide actively incorporating hydrogen (H) and deuterium (^2^H) from body water. Subsequent data handling was performed using python-based scripts, with input of precursor body water enrichment for each subject, to yield fractional synthesis rate (FSR) data at the protein level. FSR data were filtered to exclude protein measurements with fewer than 2 peptide isotope measurements per protein. Details of FSR calculations and data filtering criteria have been described in detail previously^11^.

#### Calculation of fractional replacement (f) and replacement rate constant (k) for individual proteins

Details of f calculations were previously described^11^**.**

#### Statistical analysis

Data filtering and calculations were performed according to previous reports^11^. Only those proteins that met analytic filtering criteria and that were present in at least 2 animals per group were included in comparisons and statistical analyses^19^. Individual proteins were also grouped into different functional clusters based on gene ontology origin using DAVID software from NIH website (https://david.ncifcrf.gov/tools.jsp) to determine which processes were affected at each stage^20^. Statistical significance of protein turnover comparisons was assessed by one of the following tests: (1) Global changes in mean protein turnover were compared across groups with a Student’s Unpaired T-Test, (2) a Binomial Test of the proportion of proteins showing a higher or lower value of FSR in relation to control for comparison of functional clusters across the regeneration timeline, as described previously^21^, or (3) the mean of individual proteins were compared across groups with a Student’s Unpaired T-Test with Benjamini–Hochberg correction for multiple comparisons or a One-Way ANOVA with Benjamini–Hochberg correction for multiple comparisons. Statistical significance of gene expression comparisons was assessed by one of the following tests: (1) a Binomial Test of the proportion of genes showing a higher or lower expression value in relation to control for comparison of functional clusters across the regeneration timeline, as described previously^22^, (2) correlation between mean individual protein turnover and mean individual gene expression was determined by simple linear regression equation with R^2^ value shown, or (3) the mean of individual genes were compared across groups with a Student’s Unpaired T-Test or a One-Way ANOVA with Benjamini–Hochberg correction for multiple comparisons. All statistical analysis was carried out by GraphPad Prism software (version 9.4) or Microsoft Excel (version 2021), *p < 0.05, **p < 0.01, ***p < 0.005, ****p < 0.001.

### Ethics approval and consent to participate

All mice were housed, and procedures were carried out according to the Animal Care and Use Committee (ACUC) standards in the animal facility at UC Berkeley.

## Results

### Time course and visualization of damage

**Figure 1:**
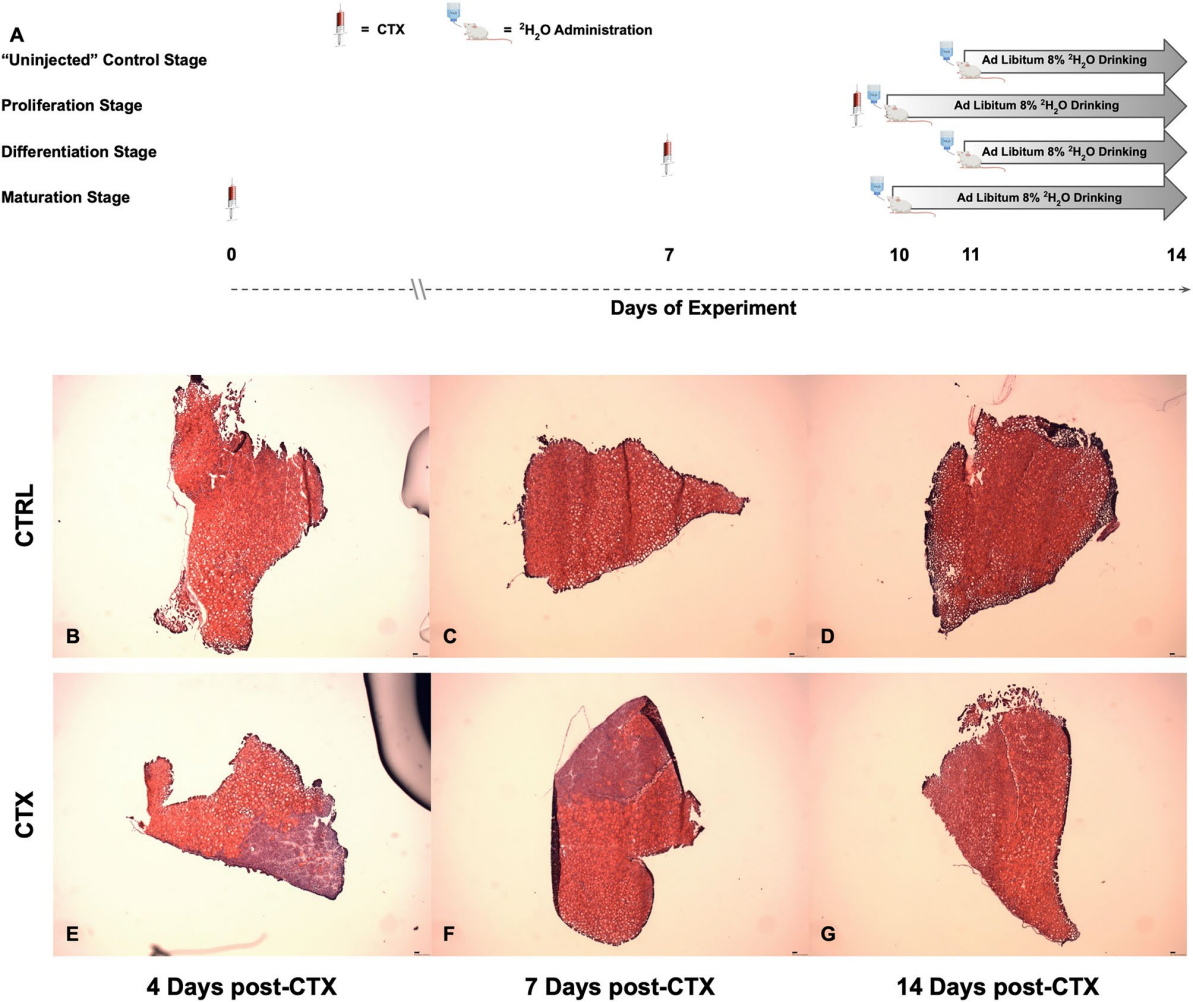
Time course layout and visualization of damage. (**A**) Overview of ^2^H_2_O labeling time course and definition of each stage based on days after CTX injection. Visualization of muscle regeneration from control muscle. (**B**) 4 days. (**C**) 7 days. (**D**) 14 days, and injured tissue. (**E**) 4 days. (**F**) 7 days. (**G**) 14 days after CTX injection. All images taken at ×5 magnification.

To induce damage and subsequent in-vivo regeneration of the skeletal muscle, we injected CTX into the Tibialis Anterior (TA) muscle of one hind limb of each mouse^23,24^. Mice injected with CTX were separated into groups differing in the timing of ^2^H_2_O labeling relative to the time of initial injury (Fig. 1A). All animals started in the protocol at the same age and time, and were sacrificed on Day 14 after CTX injection. The “Proliferation Stage” group was administered both CTX and ^2^H_2_O on the same day (Day 10 after injury) and labeled continuously until sacrifice 4 days post-CTX injection (dpi) to capture the protein flux changes that occurred during initial tissue damage, inflammation-mediated immune response, and activation/proliferation of MuSCs. The “Differentiation Stage” group was injected with CTX on Day 7 after entry, with ^2^H_2_O given from 4 to 7 dpi to capture the protein flux changes that occurred in the tissue during MuSCs differentiation into newly formed myofibers and the beginning stages of tissue regeneration. The “Maturation Stage” received a CTX injection on Day 0 and ^2^H_2_O from 10 to 14 dpi (Day 10 to Day 14 after injury) to capture the reintegration of new myofibers into existing muscle, as well as the construction of surrounding structures to support newly formed tissue. Control groups (Uninjected) received no CTX injection but were administered ^2^H_2_O for 3 days (Day 11 to Day 14 after entry). These time points were selected based on expert opinion and previous literature^2,8^. Analysis of Hematoxylin & Eosin (H&E) staining of uninjured control tissue at the Proliferation stage (Fig. 1B), Differentiation stage (Fig. 1C), and Maturation stage (Fig. 1D) showed no structural changes to the tissue. The same analysis of the CTX injured tissue at 4 dpi demonstrated widespread myofiber fragmentation and extensive immune cell infiltration at the site of CTX injection (Fig. 1E), which decreased at the beginning stages of newly formed myofibers reintegrating back into unaffected tissue was evident at 7 dpi (Fig. 1F). Visual analysis at 14 dpi shows almost complete regeneration of damaged tissue, which is almost indistinguishable from unaffected tissue (Fig. 1G). Muscle regeneration has been found to be virtually complete after 14 dpi, as previously reported^25,26^.

### DNA turnover (cell proliferation), histologic confirmation of regeneration, and expression of myogenic genes

**Figure 2:**
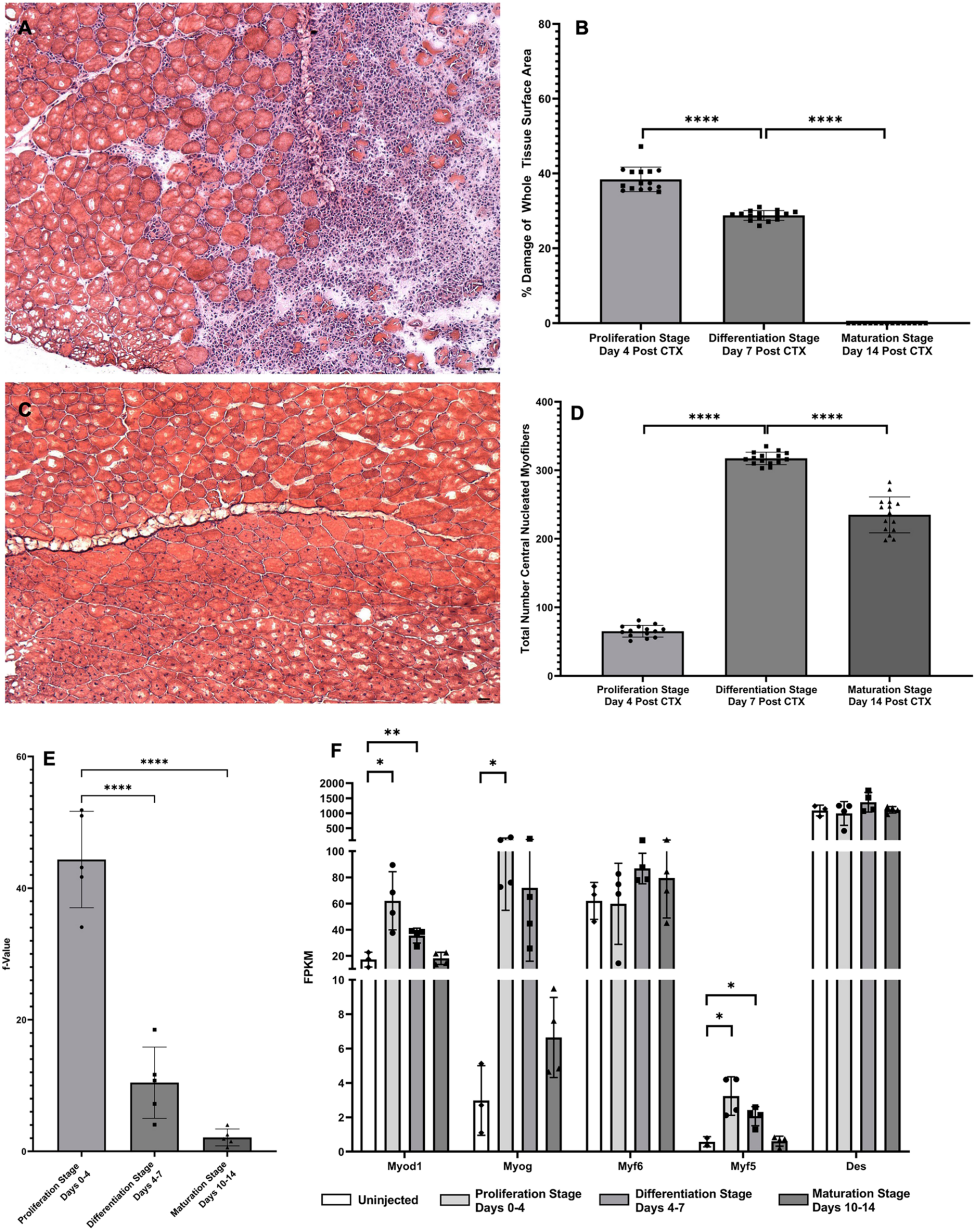
DNA turnover, visual confirmation of regeneration, and gene expression of myogenic genes. (**A**) Visualization of muscle damage 4 days after CTX injection. Image taken at ×10 magnification. (**B**) Quantification of damaged muscle tissue as a fraction of whole tissue surface area. Values are expressed as a percentage (%) of damaged tissue area in µM^2^/whole tissue surface area in µM^2^. Significance determined by One-Way ANOVA with Benjamini–Hochberg correction for multiple comparisons. *p < 0.05, **p < 0.01, ***p < 0.005, ****p < 0.001. (**C**) Visualization of central nuclei expression 14 days after CTX injection. Image taken at ×10 magnification. (**D**) Quantification of individual myofibers expressing central nuclei. Values are expressed as a total number of myofibers with central nuclei per slide. Significance determined by One-Way ANOVA with Benjamini–Hochberg correction for multiple comparisons. *p < 0.05, **p < 0.01, ***p < 0.005, ****p < 0.001. (**E**) Measuring the fractional replacement rate (f-Value) of DNA with ^2^H in newly dividing myocytes during various stages of muscle regeneration. Significance was determined by One-Way ANOVA with Benjamini Hochberg correction for multiple comparisons. *p < 0.05, **p < 0.01, ***p < 0.005, ****p < 0.001. (**F**) Myogenic gene expression in all stages was measured in FPKM and compared to control group. Significance for each individual gene determined by Student’s Unpaired T-Test, *p < 0.05, **p < 0.01, ***p < 0.005, ****p < 0.001.

Histologic quantification was carried out to confirm skeletal muscle regeneration after initial CTX damage. By H&E staining, the quantifiable fraction of damaged tissue decreased significantly (Proliferation Stage: avg. 38%, Differentiation Stage: avg 30%, p < 0.001 from Proliferation Stage; Maturation: no visible signs of damage, Fig. 2B, p < 0.001 from Proliferation Stage, p < 0.001 from Differentiation Stage) as a fraction of the entire tissue cross-sectional area (Fig. 2A). Another visual marker of skeletal muscle regeneration, myofibers with central nuclei, began forming as soon as 4 dpi, in congruence with previous findings (Fig. 2C)^26^. However, during the Differentiation Stage, the prevalence of central nuclei was significantly higher than the Proliferation Stage (Fig. 2D, p < 0.001), and decreased significantly after the Differentiation Stage transition to the Maturation Stage (Fig. 2D, p < 0.001). Along with visible quantifications of muscle regeneration, we also observed changes in cellular proliferation rates, measured by incorporation rates of ^2^H into DNA^17^. To avoid the infiltration of immune cells that proliferate around the damaged tissue in our measurements of ^2^H incorporation to DNA, single muscle fibers with attached fiber-associated cells were isolated from bulk tissue of all groups by enzymatic digestion and physical separation and then selectively depleted of leukocytes via MACS with CD45+ magnetic beads, as previously described^16^. The Proliferation Stage showed a high rate of incorporation of ^2^H in DNA, however, there was a significant drop in cellular proliferation during the Differentiation Stage and Maturation Stage in comparison to the Proliferation Stage (Proliferation Stage: avg. 44%, Differentiation Stage: avg 10%, p < 0.001 from Proliferation Stage; Maturation: avg 3%, p < 0.001 from Proliferation Stage) (Fig. 2E). These results were compared to measurements of gene expression from bulk RNA-seq values for known genes of myogenesis (Fig. 2F)^8^. At the Proliferation Stage, MyoD, MyoG, and Myf5 all showed significantly increased gene expression values, and MyoD and Myf5 still showed significant increases by the Differentiation Stage. But once the Maturation Stage was reached, there was no significant expression of myogenic genes, and expression of these genes were almost back to control levels. Genes found in bulk RNA-seq data for cell cycling (Ki67, PCNA) were also measured, and interestingly only PCNA during the Differentiation Stage was significant when compared to control (data not shown).

### Flux proteomics

To determine how protein fluxes in skeletal muscle change over time in response to damage, fractional synthesis rates (FSR) of proteins were measured in-vivo by ^2^H_2_O administration coupled with HPLC–MS/MS analysis. Since there may be a systemic response to local tissue injury^27^, we used muscle tissue from a separate mouse that was not injured as a control for most comparisons. As previously stated, we isolated single muscle fibers with attached fiber-associated cells from bulk muscle tissue by enzymatic digestion and physical separation to remove the potential influence of interstitial and other cell types from circulation on our analyses^16^.

### Comparison of global protein turnover rates at each stage

**Figure 3:**
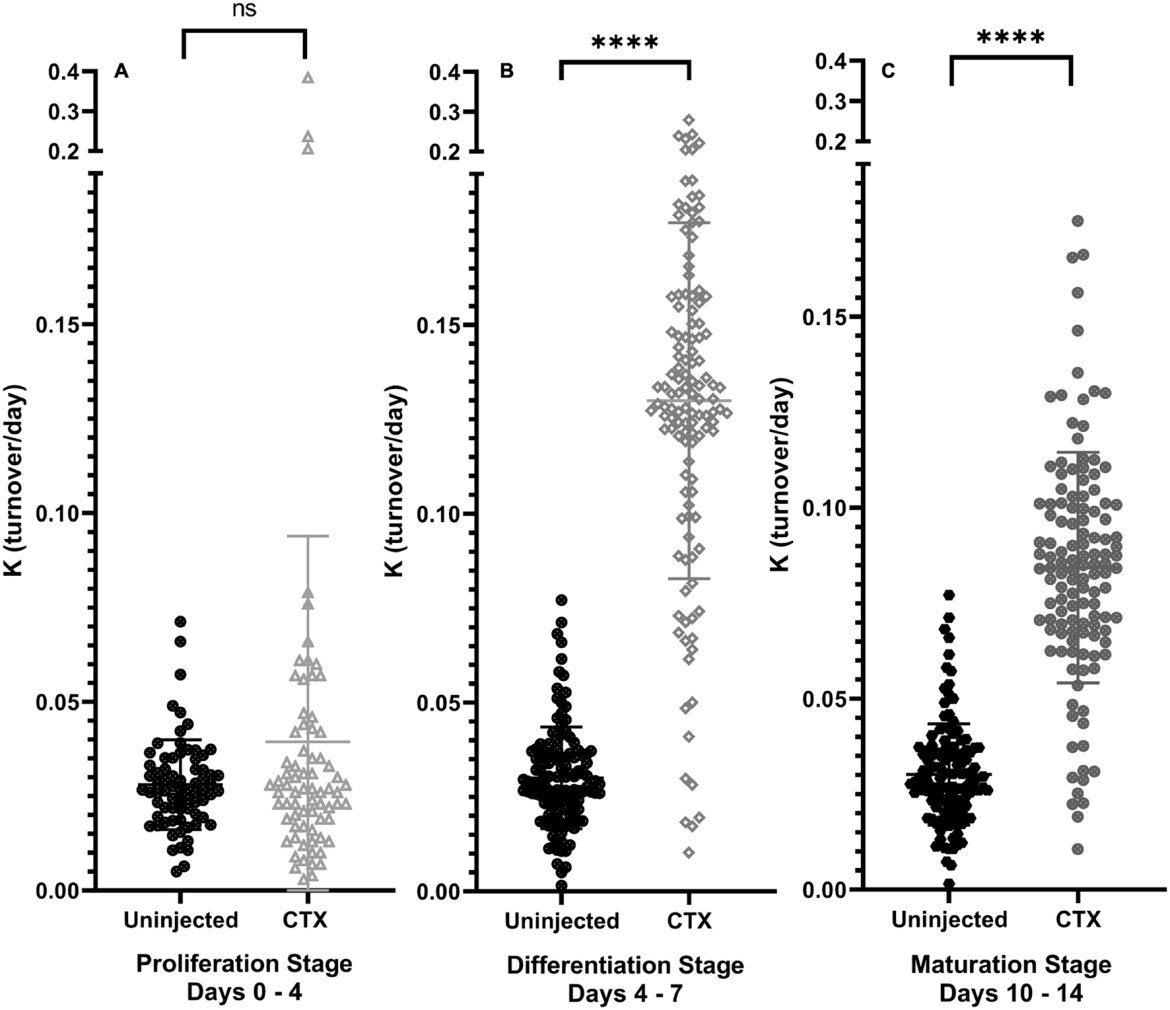
Global protein turnover rates in skeletal muscle at each stage. (**A**) Overview of global proteome FSR changes between CTX and control muscle tissue after 4 days of CTX injection. (**B**) Overview of global proteome FSR changes between CTX and control muscle tissue after 7 days of CTX injection. (**C**) Overview of global proteome FSR changes between CTX and control muscle tissue after 14 days of CTX injection. For all figures, significance determined by Student’s Unpaired T-Test, *p < 0.05, **p < 0.01, ***p < 0.005, ****p < 0.001.

At the end of the Proliferation Stage (4 dpi), the FSR of 73 proteins that passed criteria for kinetic measurements in both the CTX injured and the control limbs were compared. There was no significant global difference in protein FSR values between injured and control muscle tissue during this phase of regeneration (Fig. 3A). At the end of the Differentiation Stage, the FSR of 127 proteins that passed criteria for kinetic measurements in both the CTX injured and the control limbs were compared. Global proteome analysis shows a significant increase in protein FSR values between injured and control muscle tissue, indicating more protein flux response to injury at this stage of regeneration (p < 0.001, Fig. 3B). At the end of the Maturation Stage, the FSR of 125 proteins that passed criteria for kinetic measurements in both the CTX injured and the control limb were compared. Global proteome analysis shows a significant increase in protein FSR values between injured and control muscle tissue during this phase of regeneration (p < 0.001, Fig. 3C). Average FSR values and the standard deviation (SD) for each protein in all the stages can be found in Table 1.

**Table 1:** Protein turnover values in each stage.

#	Protein	Accession #	Ontology	Proliferation stage	Differentiation stage	Maturation stage	Control
1	Succinate dehydrogenase iron-sulfur subunit	Q9CQA3	Mitochondria	0.808 ± 0.115%*	1.954 ± 1.042%		4.721 ± 0.422%
2	Cytochrome b-c1 complex subunit 1	Q9CZ13	OxPhos	5.596 ± 0.541%*	12.412 ± 9.133%	6.976 ± 1.078%*	2.664 ± 0.422%
3	Carbonic anhydrase 3	P16015	Cytoplasm	0.670 ± 0.394%*	8.890 ± 4.592%	11.078 ± 4.185%*	2.601 ± 0.406%
4	Triosephosphate isomerase	P17751	Glycolysis	5.724 ± 1.021%*	14.663 ± 7.041%*	9.012 ± 2.761%*	2.442 ± 0.388%
5	Aconitate hydratase	Q99KI0	Mitochondria	1.635 ± 0.379%*	12.255 ± 7.028%	7.080 ± 1.327%*	3.035 ± 0.347%
6	Collagen alpha 1(III) chain	P08121	Cytoplasm	1.654 ± 0.271%*	9.080 ± 4.471%	6.479 ± 3.281%	4.899 ± 0.871%
7	Trifunctional enzyme subunit alpha	Q8BMS1	Mitochondria	0.267 ± 0.249%*	12.798 ± 9.815%	6.165 ± 3.573%	3.062 ± 1.035%
8	Histone H2B type 1-M	P10854	Histone	4.202 ± 1.405%*	6.151 ± 1.995%	3.112 ± 1.227%*	1.066 ± 0.478%
9	Phosphoglycerate kinase 1	P09411	Glycolysis	1.020 ± 0.52%	12.825 ± 6.042%	10.109 ± 3.629%*	2.604 ± 0.595%
10	NADH dehydrogenase iron-sulfur protein 6	P52503	OxPhos	2.673 ± 0.083%	8.162 ± 2.842%	6.157 ± 2.794%	3.514 ± 0.437%
11	ATP synthase subunit beta	P56480	OxPhos	0.649 ± 0.759%	13.602 ± 8.869%	7.125 ± 1.152%*	2.160 ± 0.195%
12	Myosin light chain 1/3, skeletal muscle isoform	P05977	Myofibril	2.769 ± 0.588%	18.116 ± 9.806%*	8.845 ± 2.706%*	1.703 ± 0.283%
13	Phosphatidylethanolamine-binding protein 1	P70296	Cytoplasm	23.658 ± 10.397%	17.715 ± 15.136%	7.51 ± 4.171%	3.586 ± 2.677%
14	Phosphoglucomutase-1	Q9D0F9	Glycolysis	1.329 ± 0.513%	12.066 ± 6.393%	8.285 ± 3.023%*	2.342 ± 0.399%
15	Albumin	P07724	Extracellular	1.424 ± 0.878%	13.691 ± 4.993%	15.629 ± 2.807%*	7.128 ± 3.629%
16	Myosin regulatory light chain 2, skeletal muscle isoform	P97457	Myofibril	3.144 ± 0.913%	15.749 ± 8.367%*	8.751 ± 3.235%*	1.717 ± 0.263%
17	Myosin-binding protein C, fast-type	Q5XKE0	Myofibril	20.593 ± 11.934%	22.170 ± 14.392%	12.214 ± 5.419%*	2.642 ± 0.985%
18	ATP synthase subunit g	Q9CPQ8	OxPhos	1.891 ± 0.362%	6.708 ± 3.219%	6.953 ± 1.743%*	0.644 ± 0.555%
19	L-lactate dehydrogenase A chain	P06151	Glycolysis	5.692 ± 2.301%	15.812 ± 8.103%*	10.292 ± 3.334%*	2.186 ± 0.442%
20	Hemoglobin subunit alpha	P01942	Extracellular	5.674 ± 2.723%	1.829 ± 0.147%	2.522 ± 0.502%	1.804 ± 0.491%
21	Collagen alpha 1(I) chain	P11087	Cytoplasm	2.334 ± 1.142%	6.409 ± 5.525%	4.682 ± 1.466%	3.910 ± 0.311%
22	Vertnin	Q3SYK4	Cytoplasm	1.300 ± 0.312%	1.723 ± 0.078%	1.062 ± 0.078%*	2.030 ± 0.476%
23	Glyceraldehyde-3-phosphate dehydrogenase	P16858	Glycolysis	1.221 ± 0.768%	14.165 ± 6.491%*	10.292 ± 3.231%*	2.414 ± 0.416%
24	Cytochrome c, somatic	P62897	OxPhos	0.379 ± 0.304%	12.135 ± 5.266%	8.427 ± 0.991%*	2.965 ± 0.941%
25	Calsequestrin-1	O09165	Cytoplasm	2.422 ± 1.152%	16.316 ± 10.359%	7.922 ± 2.732%*	1.067 ± 0.545%
26	Actin, alpha skeletal muscle	P68134	Myofibril	2.745 ± 1.511%	15.038 ± 7.593%	6.514 ± 1.614%*	1.136 ± 0.510%
27	Titin	A2ASS6	Myofibril	1.257 ± 0.947%	7.304 ± 5.401%	2.240 ± 1.205%	2.388 ± 0.611%
28	Malate dehydrogenase	P08249	Mitochondria	38.498 ± 31.447%	11.888 ± 7.637%	6.811 ± 1.574%*	2.171 ± 0.412%
29	Myosin light chain 3	P09542	Myofibril	2.029 ± 0.489%	13.340 ± 11.791%	8.709 ± 3.725%	3.654 ± 1.621%
30	ADP/ATP translocase 1	P48962	Mitochondria	2.271 ± 0.783%	20.589 ± 9.866%	9.328 ± 1.604%*	3.912 ± 1.588%
31	Parvalbumin alpha	P32848	Extracellular	7.899 ± 3.726%	12.598 ± 10.416%	11.055 ± 2.638%*	4.406 ± 1.471%
32	Aspartate aminotransferase	P05201	Cytoplasm	7.616 ± 5.416%	9.385 ± 4.313%	6.651 ± 4.126%	2.419 ± 0.991%
33	NADH dehydrogenase 1 beta subcomplex subunit 10	Q9DCS9	OxPhos	0.678 ± 0.197%	14.674 ± 7.989%	6.729 ± 1.887%*	1.739 ± 0.895%
34	Glycogen phosphorylase, muscle form	Q9WUB3	Glycolysis	3.023 ± 0.667%	18.813 ± 7.782%	12.908 ± 3.501%*	3.732 ± 0.561%
35	Myozenin-1	Q9JK37	Myofibril	6.098 ± 2.751%	4.098 ± 2.064%	2.262 ± 1.449%	3.517 ± 1.355%
36	Pyruvate kinase PKM	P52480	Glycolysis	4.596 ± 1.726%	15.914 ± 7.028%	11.808 ± 3.548%*	3.038 ± 0.533%
37	Myoglobin	P04247	Extracellular	2.635 ± 0.248%	12.661 ± 6.126%	9.071 ± 3.379%*	3.216 ± 0.700%
38	Fructose-bisphosphate aldolase A	P05064	Glycolysis	2.848 ± 0.989%	17.324 ± 6.718%	14.635 ± 4.516%*	3.739 ± 0.572%
39	Parkinson disease protein 7 homolog	Q99LX0	Cytoplasm	4.725 ± 3.626%	9.929 ± 2.413%	8.499 ± 4.634%*	1.454 ± 0.852%
40	Troponin T, fast skeletal muscle	Q9QZ47	Myofibril	2.339 ± 1.719%	23.964 ± 10.275%	12.996 ± 3.924%*	3.483 ± 0.242%
41	LIM domain-binding protein 3	Q9JKS4	Cytoplasm	2.589 ± 0.743%	13.857 ± 6.588%*	7.606 ± 2.068%*	1.676 ± 1.034%
42	Actin, alpha cardiac muscle 1	P68033	Myofibril	2.162 ± 1.218%	15.755 ± 7.358%	6.709 ± 1.859%*	1.310 ± 0.585%
43	Histone H4	P62806	Histone	4.315 ± 1.31%	8.854 ± 3.891%	**3.732 ± 1.013%**	6.602 ± 3.354%
44	Sarcoplasmic/endoplasmic reticulum calcium ATPase 1	Q8R429	Cytoplasm	3.372 ± 0.913%	12.162 ± 6.198%	11.181 ± 2.325%*	2.817 ± 0.330%
45	Alpha-actinin-3	O88990	Myofibril	6.104 ± 5.176%	18.834 ± 16.162%	9.670 ± 2.019%*	3.317 ± 0.560%
46	Phosphoglycerate mutase 2	O70250	Glycolysis	1.863 ± 0.543%	12.236 ± 5.528%*	9.897 ± 3.362%*	2.199 ± 0.390%
47	Cytochrome b-c1 complex subunit 7	Q9D855	OxPhos	5.99 ± 4.283%	14.071 ± 10.397%	9.180 ± 4.145%	3.208 ± 0.168%
48	ATP synthase subunit O	Q9DB20	OxPhos	0.933 ± 0.428%	12.058 ± 6.922%	8.408 ± 1.559%*	1.938 ± 1.097%
49	ATP synthase subunit d	Q9DCX2	OxPhos	2.636 ± 2.595%	14.703 ± 10.311%	8.299 ± 0.921%*	4.226 ± 1.229%
50	Tropomyosin beta chain	P58774	Myofibril	3.483 ± 1.545%	20.450 ± 9.492%	9.634 ± 2.737%*	2.7645 ± 0.676%
51	Aspartate aminotransferase	P05202	Mitochondria	3.321 ± 1.854%	13.195 ± 9.701%	5.799 ± 0.211%*	1.958 ± 1.162%
52	Dihydrolipoyl dehydrogenase	O08749	Mitochondria	4.188 ± 1.905%	15.802 ± 9.119%	10.881 ± 1.704%*	5.727 ± 1.009%
53	ADP/ATP translocase 2	P51881	Mitochondria	2.256 ± 1.237%	12.591 ± 3.295%*	7.058 ± 2.584%	3.200 ± 1.467%
54	Glucose-6-phosphate isomerase	P06745	Glycolysis	2.091 ± 1.296%	14.763 ± 6.794%	9.576 ± 3.205%*	1.549 ± 0.269%
55	Nucleoside diphosphate kinase B	Q01768	Cytoplasm	3.518 ± 1.34%	10.913 ± 4.194%	3.092 ± 0.888%	2.918 ± 0.759%
56	Myosin-3	P13541	Myofibril	3.098 ± 0.502%	17.741 ± 6.788%*	8.632 ± 2.026%*	2.845 ± 0.454%
57	Creatine kinase M-type	P07310	Cytoplasm	2.088 ± 0.683%	12.766 ± 7.031%	10.725 ± 3.614%*	2.334 ± 0.334%
58	Adenylate kinase isoenzyme 1	Q9R0Y5	Cytoplasm	6.56 ± 6.691%	13.356 ± 8.524%	10.091 ± 3.218%	2.676 ± 1.245%
59	Pyruvate dehydrogenase E1 component subunit beta	Q9D051	Mitochondria	1.402 ± 0.531%	17.912 ± 17.817%	10.069 ± 3.588%*	1.7367 ± 0.485%
60	Centrosomal protein of 162 kDa	Q6ZQ06	Cytoplasm	0.998 ± 0.807%	1.029 ± 0.223%		0.503 ± 0.101%
61	Troponin C, skeletal muscle	P20801	Myofibril	4.432 ± 2.101%	23.207 ± 12.899%	12.944 ± 0.351%*	3.645 ± 0.702%
62	Myosin-7	Q91Z83	Myofibril	2.919 ± 0.552%	19.078 ± 8.624%	10.105 ± 3.507%*	2.692 ± 0.692%
63	NADH-ubiquinone oxidoreductase 75 kDa subunit	Q91VD9	OxPhos	2.253 ± 1.163%	13.502 ± 9.761%	9.697 ± 0.154%*	2.645 ± 1.129%
64	Tropomyosin alpha-1 chain	P58771	Myofibril	3.292 ± 1.068%	24.316 ± 11.703%*	12.135 ± 3.619%*	3.051 ± 0.435%
65	Desmoglein-4	Q7TMD7	Extracellular	2.278 ± 0.358%	6.637 ± 1.868%	6.252 ± 1.573%*	2.543 ± 1.875%
66	Troponin I, fast skeletal muscle	P13412	Myofibril	2.772 ± 3.786%	14.408 ± 9.648%	11.275 ± 3.391%*	3.120 ± 0.638%
67	ATP synthase subunit alpha	Q03265	OxPhos	3.069 ± 2.453%	13.685 ± 8.443%	7.798 ± 0.997%*	2.821 ± 0.628%
68	Beta-enolase	P21550	Glycolysis	2.993 ± 0.806%	15.768 ± 6.887%	9.799 ± 2.771%*	2.928 ± 0.528%
69	Myosin-4	Q5SX39	Myofibril	2.617 ± 0.525%	13.03 ± 6.334%	8.540 ± 2.271%*	2.579 ± 0.395%
70	Collagen alpha-2(I) chain	Q01149	Cytoplasm	1.749 ± 0.882%	6.855 ± 5.648%	4.358 ± 0.173%	1.853 ± 0.134%
71	Cytochrome b-c1 complex subunit 2	Q9DB77	OxPhos	3.741 ± 0.687%	12.696 ± 8.177%	7.910 ± 1.345%*	3.716 ± 0.187%
72	Myosin-1	Q5SX40	Myofibril	2.662 ± 0.771%	18.191 ± 10.073%*	7.138 ± 1.637%*	2.675 ± 0.572%
73	Superoxide dismutase [Mn]	P09671	Mitochondria	1.881 ± 0.811%	7.240 ± 5.53%	5.747 ± 2.585%*	1.869 ± 0.356%
74	Heat shock cognate 71 kDa protein	P63017	Cytoplasm		18.396 ± 6.43%	9.970 ± 0.024%*	5.112 ± 2.252%
75	Alpha-enolase	P17182	Glycolysis		11.379 ± 3.973%	7.056 ± 2.348%*	3.629 ± 0.958%
76	Histone H2A type 1-B	C0HKE1	Histone		5.011 ± 1.761%	1.908 ± 0.636%*	0.155 ± 0.068%
77	Ubiquinone biosynthesis protein COQ9	Q8K1Z0	Mitochondria		11.913 ± 4.233%*	10.460 ± 3.135%*	2.646 ± 0.885%
78	E3 ubiquitin-protein ligase UBR4	A2AN08	Cytoplasm		13.175 ± 5.817%*	7.763 ± 2.892%*	2.687 ± 0.388%
79	E3 ubiquitin-protein ligase UBR3	Q5U430	Cytoplasm		27.949 ± 15.189%*	17.507 ± 7.163%*	1.223 ± 1.009%
80	Very long-chain specific acyl-CoA dehydrogenase	P50544	Mitochondria		16.838 ± 7.417%	8.786 ± 3.464%	3.402 ± 1.956%
81	Creatine kinase S-type	Q6P8J7	Mitochondria		7.134 ± 2.504%	8.734 ± 2.252%*	2.673 ± 0.425%
82	Cytochrome c oxidase subunit 6C	Q9CPQ1	OxPhos		14.812 ± 6.971%	**7.436 ± 3.237%**	2.863 ± 0.368%
83	Sarcalumenin	Q7TQ48	Cytoplasm		13.037 ± 7.622%	6.788 ± 2.051%*	1.861 ± 0.849%
84	NADH dehydrogenase flavoprotein 1	Q91YT0	OxPhos		13.358 ± 6.28%	8.120 ± 2.029%*	2.638 ± 0.931%
85	Keratin, type II cytoskeletal 1	P04104	Cytoplasm		12.781 ± 2.845%	8.523 ± 3.349%	6.156 ± 2.426%
86	Voltage-dependent anion-selective channel protein 2	Q60930	Mitochondria		17.969 ± 10.852%	16.622 ± 9.964%*	1.172 ± 0.341%
87	2-oxoglutarate dehydrogenase	Q60597	Mitochondria		12.728 ± 4.676%	**10.484 ± 0.553%**	4.590 ± 2.8085%
88	MICOS complex subunit Mic60	Q8CAQ8	Mitochondria		12.908 ± 6.049%	13.049 ± 3.866%*	2.017 ± 0.955%
89	ATP synthase-coupling factor 6	P97450	OxPhos		13.338 ± 8.724%	4.843 ± 1.787%*	0.727 ± 0.437%
90	Cytochrome c oxidase subunit 4 isoform 1	P19783	OxPhos		13.407 ± 6.626%	9.101 ± 1.772%*	3.773 ± 1.137%
91	Isocitrate dehydrogenase [NAD] subunit alpha	Q9D6R2	OxPhos		12.691 ± 6.118%	**5.350 ± 2.844%**	3.388 ± 1.499%
92	Malate dehydrogenase	P14152	Cytoplasm		10.222 ± 6.133%	9.054 ± 3.799%*	3.049 ± 0.749%
93	Citrate synthase	Q9CZU6	Mitochondria		13.995 ± 8.577%	8.780 ± 1.445%*	2.581 ± 0.272%
94	Cytochrome c oxidase subunit NDUFA4	Q62425	OxPhos		19.203 ± 9.895%	13.529 ± 3.471%*	4.204 ± 1.703%
95	Voltage-dependent anion-selective channel protein 1	Q60932	Mitochondria		12.654 ± 8.096%	10.117 ± 2.361%*	2.609 ± 0.838%
96	Cytochrome c oxidase subunit 2	P00405	OxPhos		12.272 ± 9.047%	6.237 ± 1.392%*	1.093 ± 0.249%
97	Cytochrome c oxidase subunit 6B1	P56391	OxPhos		15.028 ± 10.416%	8.420 ± 1.31%*	2.232 ± 0.255%
98	Alpha-actinin-2	Q9JI91	Myofibril		15.588 ± 9.833%	8.145 ± 1.074%*	3.641 ± 0.774%
99	Cytochrome c oxidase subunit 5B	P19536	OxPhos		13.394 ± 9.383%	7.503 ± 1.776%*	2.547 ± 0.453%
100	Myelin protein P0	P27573	Cytoplasm		9.877 ± 7.41%	9.217 ± 2.874%*	2.321 ± 1.902%
101	NADH dehydrogenase 1 beta subcomplex subunit 1	P0DN34	OxPhos		16.550 ± 11.136%	8.989 ± 1.226%*	3.945 ± 0.583%
102	Cytochrome b-c1 complex subunit Rieske	Q9CR68	OxPhos		12.612 ± 8.751%	8.267 ± 1.666%*	2.817 ± 0.305%
103	ATP synthase subunit gamma	Q91VR2	OxPhos		12.435 ± 8.037%	6.224 ± 1.147%*	1.698 ± 0.066%
104	Cytochrome c1, heme protein	Q9D0M3	OxPhos		11.024 ± 7.245%	6.761 ± 0.793%*	3.057 ± 0.585%
105	Cytochrome b-c1 complex subunit 6	P99028	OxPhos		12.398 ± 9.641%	5.770 ± 0.133%*	1.871 ± 0.452%
106	Cytochrome b-c1 complex subunit 8	Q9CQ69	OxPhos		14.625 ± 10.285%	9.990 ± 0.663%	4.041 ± 0.685%
107	Elongation factor 1-alpha 2	P62631	Cytoplasm		17.518 ± 14.891%	10.873 ± 2.597%*	4.156 ± 2.371%
108	NADH dehydrogenase [ubiquinone] 1 alpha subcomplex subunit 7	Q9Z1P6	OxPhos		18.110 ± 12.195%	11.246 ± 1.436%*	5.817 ± 0.602%
109	Cytochrome c oxidase subunit 5A	P12787	OxPhos		14.048 ± 10.802%	8.566 ± 0.965%*	3.395 ± 0.516%
110	NADH dehydrogenase 1 alpha subcomplex subunit 8	Q9DCJ5	OxPhos		15.487 ± 12.898%	11.041 ± 1.409%*	3.267 ± 0.563%
111	Triadin	E9Q9K5	Cytoplasm		15.377 ± 13.226%	8.131 ± 1.473%*	3.556 ± 0.703%
112	Calcium-binding mitochondrial carrier protein Aralar1	Q8BH59	Mitochondria		10.569 ± 5.878%	8.784 ± 2.103%*	2.452 ± 1.022%
113	NADH dehydrogenase iron-sulfur protein 3	Q9DCT2	OxPhos		7.959 ± 4.907%	7.905 ± 3.656%	3.710 ± 0.145%
114	NADH dehydrogenase 1 beta subcomplex subunit 9	Q9CQJ8	OxPhos		13.572 ± 10.199%	2.870 ± 1.765%	3.540 ± 1.634%
115	ATP-dependent 6-phosphofructokinase, muscle type	P47857	Glycolysis		12.190 ± 10.024%	16.549 ± 4.776%*	5.001 ± 1.547%
116	NADH dehydrogenase iron-sulfur protein 5	Q99LY9	OxPhos		13.174 ± 10.451%	7.292 ± 1.487%*	3.188 ± 0.748%
117	NADH dehydrogenase iron-sulfur protein 8	Q8K3J1	OxPhos		14.293 ± 13.812%	6.928 ± 3.745%	1.944 ± 0.159%
118	Succinate dehydrogenase flavoprotein subunit	Q8K2B3	Mitochondria		10.575 ± 5.816%	7.172 ± 3.902%	5.272 ± 2.281%
119	ATP synthase subunit delta	Q9D3D9	OxPhos		4.852 ± 3.366%	3.765 ± 1.253%	1.343 ± 0.017%
120	NADH dehydrogenase flavoprotein 2	Q9D6J6	OxPhos		7.424 ± 5.079%	4.547 ± 0.277%	3.601 ± 0.433%
121	ATP synthase F (0) complex subunit B1	Q9CQQ7	OxPhos		12.538 ± 8.688%	7.485 ± 1.505%	6.824 ± 6.658%
122	Glycerol-3-phosphate dehydrogenase	Q64521	Mitochondria		12.417 ± 9.942%	11.004 ± 2.957%	4.086 ± 1.487%
123	Cytochrome c oxidase subunit 6A2	P43023	OxPhos		18.43 ± 11.419%	12.832 ± 3.977%	7.717 ± 3.418%
124	NADH dehydrogenase 1 alpha subcomplex subunit 9	Q9DC69	OxPhos		9.906 ± 5.739%	8.373 ± 1.546%	5.375 ± 1.076%
125	Hemoglobin subunit beta 1	P02088	Extracellular		2.987 ± 0.566%	2.933 ± 0.685%	2.598 ± 0.668%
126	Electron transfer flavoprotein subunit alpha	Q99LC5	OxPhos		8.782 ± 9.531%	9.228 ± 2.616%	4.549 ± 0.808%
127	Phosphate carrier protein	Q8VEM8	Mitochondria		2.824 ± 1.441%	6.119 ± 3.707%	3.548 ± 2.193%

Average % turnover per day ± standard deviation of each individual protein in each group. Proteins with bold text and a * in their boxes are significant by Student’s Unpaired T-Test with Benjamini Hochberg correction for multiple comparisons when compared to their control value, *p < 0.05. OxPhos = oxidative phosphorylation.

### Changes in protein ontology group turnover rates in each stage

**Figure 4:**
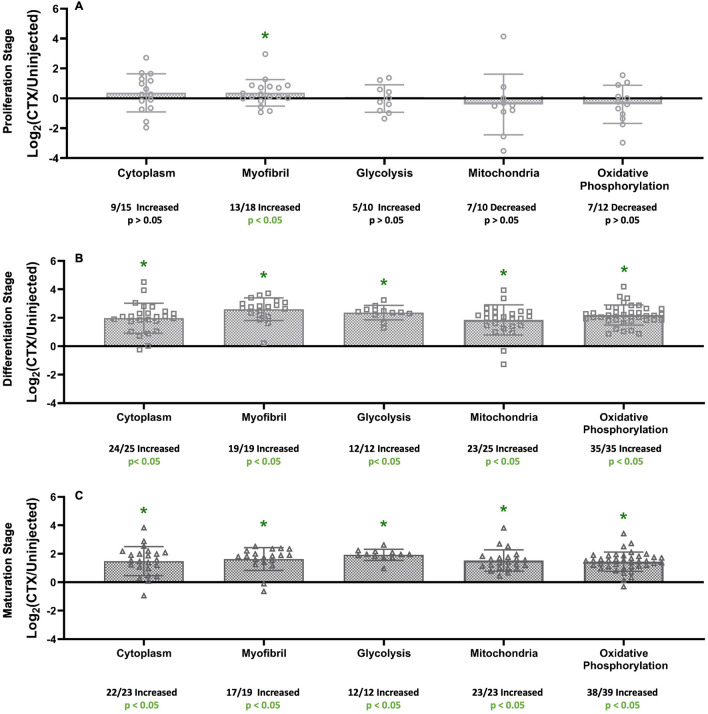
Changes in protein group turnover rates in each stage. (**A**) Protein Group analysis of proteins found during the Proliferation Stage (4 dpi) and separated into ontologies based on NIH DAVID analysis. (**B**) Protein Group analysis of proteins found during the Differentiation Stage (7 dpi) and separated into ontologies based on NIH DAVID analysis. (**C**) Protein Group analysis of proteins found during the Maturation Stage (14 dpi) and separated into ontologies based on NIH DAVID analysis. Significance determined by Binomial Test of the proportion of proteins showing a higher or lower value of FSR in relation to uninjected (control), *p < 0.05.

Functional clusters of protein groups were analyzed by gene ontology enrichment using DAVID software from NIH to determine which cellular processes were highly affected at each stage (Fig. 4A–C). In the Proliferation Stage, only the Myofibril cluster had a significant number of proteins with higher FSR values in the injured limb compared to control limb (13/18 increased, p < 0.05). Other functional clusters [Cytoplasm (9/15 increased, p > 0.05), Glycolysis (5/10 increased, p > 0.05), Mitochondria (7/10 decreased, p > 0.05), and Oxidative Phosphorylation (7/12 decreased, p > 0.05)] were not significantly affected in this stage. In the Differentiation Stage, all measured functional clusters [Cytoplasm (24/25 increased, p < 0.05), Myofibril (19/19 increased, p < 0.05), Glycolysis (12/12 increased, p < 0.05), Mitochondria (24/25 increased, p < 0.05), and Oxidative Phosphorylation (35/35 increased, p < 0.05)] had a significant increase in the number of proteins with a higher FSR value in the injured limb compared to control limb (Fig. 4B). In the Maturation Stage, all measured functional cluster protein groups [Cytoplasm (22/23 increased, p < 0.05), Myofibril (17/19 increased, p < 0.05), Glycolysis (12/12 increased, p < 0.05), Mitochondria (23/23 increased, p < 0.05), and Oxidative Phosphorylation (38/39 increased, p < 0.05)] showed significant increases in the number of proteins with a higher FSR value in the injured limb compared to control limb (Fig. 4C).

### Individual protein turnover rates at each stage

**Figure 5:**
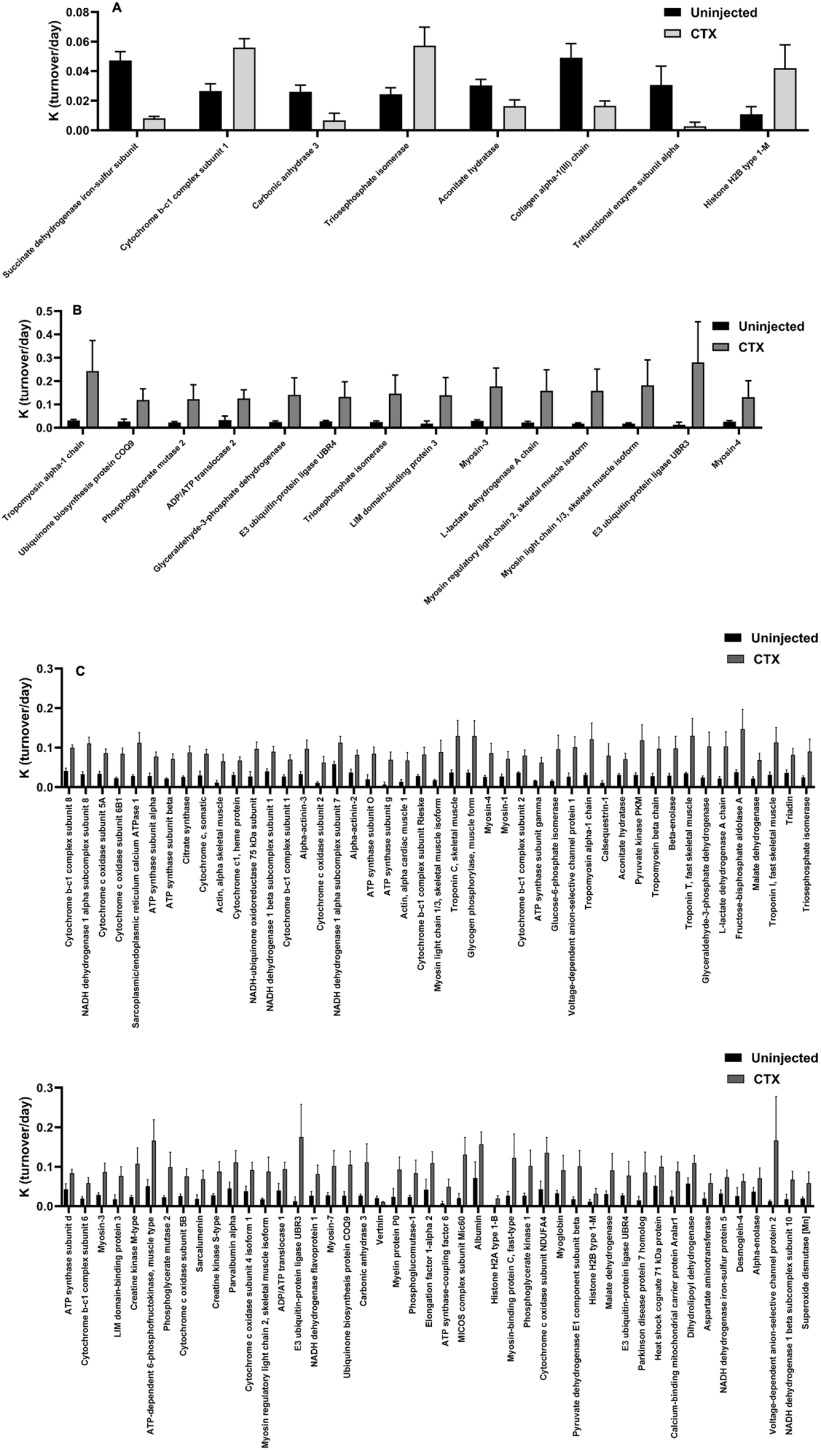
Significantly different individual protein turnover rates at each stage. (**A**) Comparison of change in individual protein FSR value between CTX injected and control limbs for proteins that are significantly different after Benjamini Hochberg correction for multiple comparisons at 4 days after CTX injection (Proliferation Stage). (**B**) Comparison of change in individual protein FSR value between CTX injected and control limbs for proteins that are significantly different after Benjamini Hochberg correction for multiple comparisons at 7 days after CTX injection (Differentiation Stage). (**C**) Comparison of change in individual protein FSR value between CTX injected and control limbs for proteins that are significantly different after Benjamini Hochberg correction for multiple comparisons at 14 days after CTX injection (Maturation Stage). Significance determined by Student’s Unpaired T-Test with Benjamini Hochberg correction for multiple comparisons, *p < 0.05, **p < 0.01, ***p < 0.005, ****p < 0.001.

In the Proliferation Stage, 8 individual proteins stood out as statistically significant after correction for multiple comparisons between CTX injected and control muscle (Fig. 5A). Proteins from skeletal muscle that have been previously identified as systemic markers of whole muscle FSR values (CA3)^28^, proteins involved in accessing cellular components to DNA to either promote or silence the DNA replication processes (Histone H2B Type 1-M)^29^, and individual glycolytic enzymes, like TPI, showed a significant change in FSR during this stage of regeneration. In the Differentiation Stage, 14 individual proteins were significant after correction for multiple comparisons that include different Myosin Heavy Chain isoforms including Myosin 3, also known as Embryonic Myosin Heavy Chain (EmyHC) and Myosin Light Chain 1/3^30–32^. A few more glycolytic enzymes than just TPI (Phosphoglycerate Mutase 2 (PGM-2), Glyceraldehyde-3-dehydrogenase (G3PDH), & Lactate Dehydrogenase A (LDH-A)) had significantly increased FSR values during this stage of regeneration as well (Fig. 5B). In the Maturation Stage, 91 individual proteins stood out as having significantly different FSR values in the injured limb compared to control limb after correction for multiple comparisons (Fig. 5C). Previously mentioned individual proteins of interest, (CA3, as well as Creatine Kinase-Muscle isoform (CK-M), and Myosin Light Chain 1/3) had significantly higher protein turnover at this stage of regeneration as well^28^. Interestingly, Myosin 3 also had a significantly higher FSR in the injured muscle at this time point, after nascent myofibers began to mature^31^. Also, Histone H2B Type 1-M is seen to have a significant increase in this stage as well. All measured glycolysis proteins, including the ones mentioned above (TPI, PGM-2, G3PDH, LDH-A), were significantly increased as well.

### Global gene expression changes at each stage

**Figure 6:**
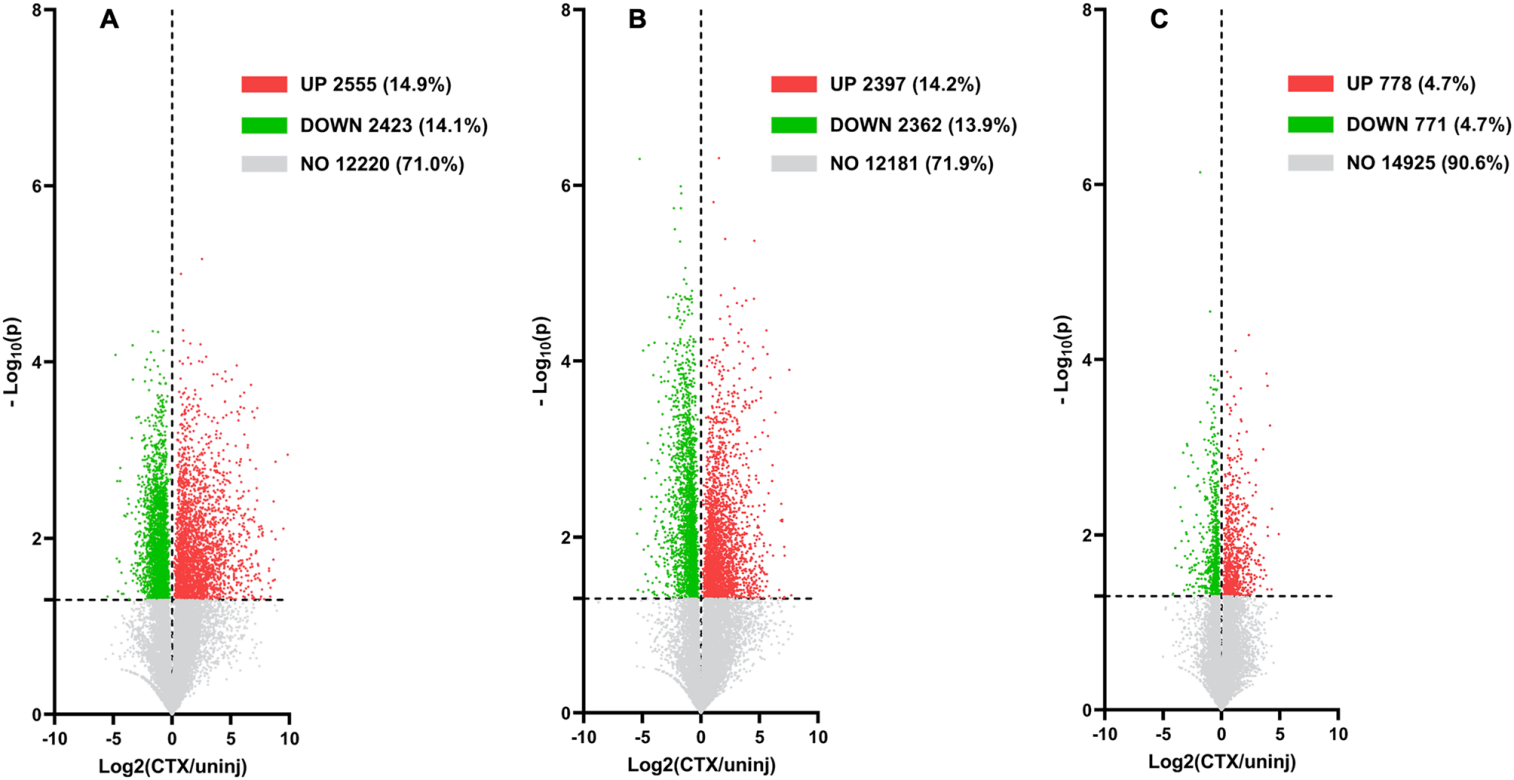
Global gene expression rates at each stage. (**A**) Volcano plot of global gene expression (FPKM) on the X-axis and Log_10_(p value) on the Y-axis for all genes found during the Proliferation Stage (4 dpi). (**B**) Volcano plot of global gene expression (FPKM) on the X-axis and Log_10_(p value) on the Y-axis for all genes found during the Differentiation Stage (7 dpi). (**C**) Volcano plot of global gene expression (FPKM) on the X-axis and Log_10_(p value) on the Y-axis for all genes found during the Maturation Stage (14 dpi). All values with a -log10(p value) of below 1.301 were non-significant and are colored gray.

Gene expression values were normalized to fragment per kilobase millions (FPKM) values, which were used to compare global gene expression profiles of each stage of muscle regeneration. In the Proliferation Stage, 17,198 individual gene expression values were measured. Of those 17,198 genes, 2555 genes (14.9%) were significantly upregulated, 2423 genes (14.1%) were significantly downregulated, and 12,220 (71.0%) genes did not change significantly (-Log_10_(p) > 1.301, Fig. 6A). In the Differentiation Stage, 16,940 individual gene expression values were measured. Of those 16,940 genes, 2397 genes (14.2%) were significantly upregulated, 2362 genes (13.9%) were significantly downregulated, and 12,181 genes (71.9%) did not change significantly (−Log_10_(p) > 1.301, Fig. 6B). In the Maturation Stage, 16,474 individual gene expression values were measured. Of those 16,474 genes, 778 genes (4.7%) were significantly upregulated, 771 genes (4.7%) were significantly downregulated, and 14,925 genes (90.6%) did not change significantly (−Log_10_(p) > 1.301, Fig. 6C). Up to the Top 50 Gene Ontology Biological Processes (GOBP’s) were measured from the list of genes that were either significantly upregulated or downregulated in each stage and can be viewed in the Supplemental Data (Figs. S1–S3).

### Group ontology gene expression at each stage for matched proteins

**Figure 7:**
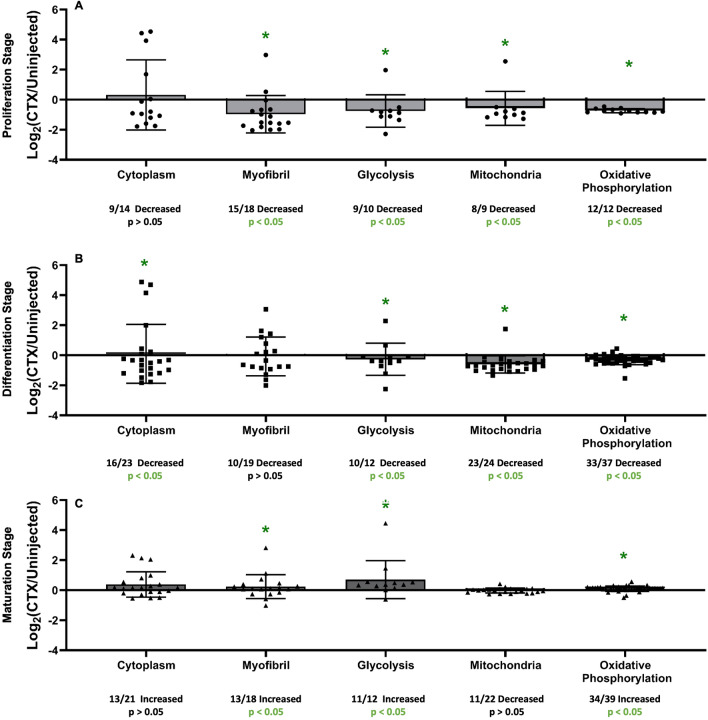
Gene expression at each stage for matched individual proteins in group ontologies. (**A**) Functional Clusters of gene expression based on protein groups (used in Fig. 4) from NIH DAVID software at the Proliferation Stage (4 dpi). (**B**) Functional Clusters of gene expression based on protein groups (used in Fig. 4) from NIH DAVID software at the Differentiation Stage (7 dpi). (**C**) Functional Clusters of gene expression based on protein groups (used in Fig. 4) from NIH DAVID software at the Maturation Stage (14 dpi). For all groups, significance is determined by Binomial Test of the proportion of proteins showing a higher or lower value of FSR in relation to control, *p < 0.05.

UniProt unique protein identification numbers (Accession #) were used to find Gene IDs for all proteins that were measured in functional cluster protein groups from DAVID by NIH software. FPKM values for each of those individual genes identified were ratioed to generate a Log_2_(Fold Change) to measure the magnitude of change between each stage of muscle regeneration and control tissue. These values were then split up into the same functional cluster protein groups, as seen in Fig. 4. In the Proliferation Stage, all of the functional clusters that were measured had a significant number of proteins with lower FSR values in the injured limb compared to control limb [Myofibril (15/18 decreased, p < 0.05), Glycolysis (9/10 decreased, p < 0.05), Mitochondria (8/9 decreased, p < 0.05), and Oxidative Phosphorylation (12/12 decreased, p < 0.05)], except for Cytoplasm (9/14 decreased, p > 0.05) (Fig. 7A). In the Differentiation Stage, again most of the functional clusters that were measured had a significant number of proteins with lower FSR values in the injured limb compared to control limb [Cytoplasm (16/23 decreased, p < 0.05), Glycolysis (10/12 decreased, p < 0.05), Mitochondria (23/24 decreased, p < 0.05), and Oxidative Phosphorylation (33/37 decreased, p < 0.05)], except for Myofibril (10/19 decreased, p > 0.05), (Fig. 7B). In the Maturation Stage, 3 of the measured functional cluster protein groups [Myofibril (13/18 increased, p < 0.05), Glycolysis (11/12 increased, p < 0.05), Oxidative Phosphorylation (34/39 increased, p < 0.05)] saw significant increases in the number of proteins with a higher FSR value in the injured limb compared to control limb, while 2 did not [Cytoplasm (13/21 increased, p > 0.05), Mitochondria (11/22 decreased, p > 0.05)] (Fig. 7C).

### Correlations of changes in gene expression to changes in protein FSR

**Figure 8:**
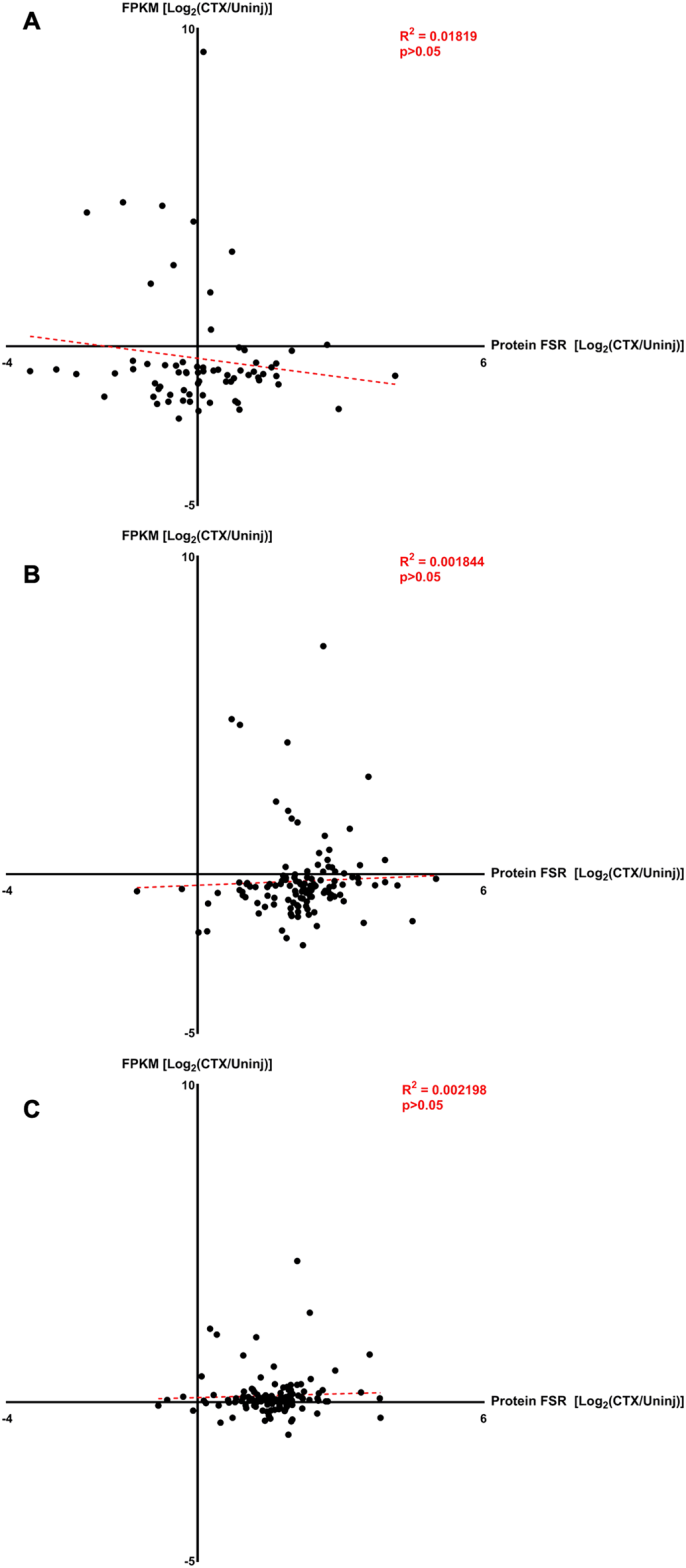
Correlation of gene expression to protein FSR. (**A**) Correlation graphs between log transformed fold change in gene expression (FPKM, Y Axis) and log transformed fold change in protein turnover (FSR, X Axis) for Proliferation Stage (4 dpi). (**B**) Correlation graphs between log transformed fold change in gene expression (FPKM, Y Axis) and log transformed fold change in protein turnover (FSR, X Axis) for Differentiation Stage (7 dpi). (**C**) Correlation graphs between log transformed fold change in gene expression (FPKM, Y Axis) and log transformed fold change in protein turnover (FSR, X Axis) for Maturation Stage (14 dpi). For all graphs, significance was determined by simple linear regression equation with R^2^ value shown.

To correlate changes in gene expression FPKM values with their protein FSR values, a 2-dimensional graph was created to plot the Log_2_FC of FPKM values on the Y axis and Log_2_FC of protein FSR values on the X axis for each stage of muscle regeneration. In the Proliferation Stage, there is no significant correlation between the magnitude of change in FPKM and FSR values (R^2^ = 0.01819, p > 0.05, Fig. 8A). In the Differentiation Stage, there is also no significant correlation between the magnitude of change in FPKM and FSR values (R^2^ = 0.00184, p > 0.05, Fig. 8B). In the Maturation Stage as well, there is no significant correlation between the magnitude of change in FPKM and FSR values (R^2^ = 0.00220, p > 0.05, Fig. 8C). These results indicate that there is no significant correlation between the magnitude of change in gene expression values and the magnitude of change in protein turnover at any of the stages of muscle regeneration.

### Myh3 gene expression, EmyHC protein abundance, EmyHC protein levels, and My3 protein turnover

**Figure 9:**
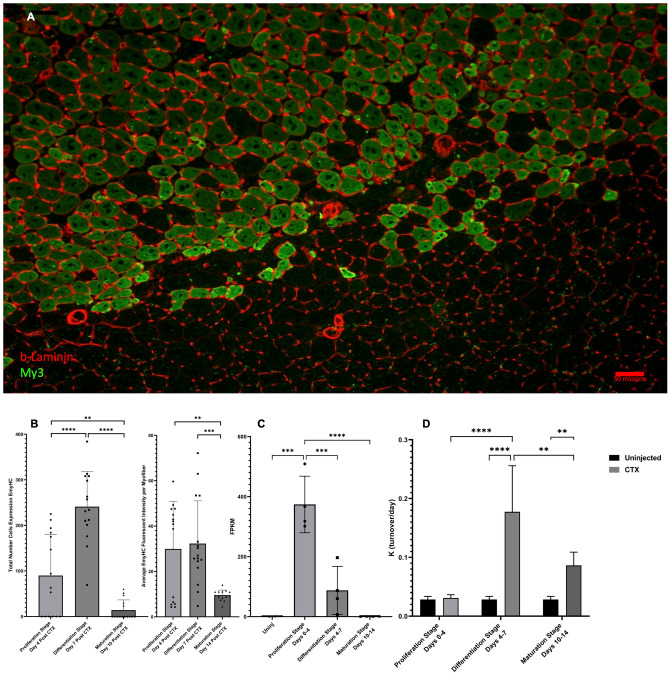
Myh3 gene expression, EmyHC protein abundance, and My3 protein turnover. (**A**) Visualization of EmyHC (green) expression, counterstained with β-Laminin (red), at 7 days post CTX injection (Differentiation Stage). (**B**) Quantification of individual myofibers expressing Embryonic Myosin Heavy Chain (EmyHC). Values are expressed as: number of myofibers/100 µM^2^ of tissue. Average measurement of protein levels by fluorescent staining intensity of individual myofibers at each stage. Significance for both graphs was determined by One-Way ANOVA with Benjamini–Hochberg correction for multiple comparisons. *p < 0.05, **p < 0.01, ***p < 0.005, ****p < 0.001. (**C**) Gene expression (FPKM) of Myh3 during various stages of muscle regeneration. Significance determined by One-Way ANOVA with Benjamini–Hochberg correction for multiple comparisons. *p < 0.05, **p < 0.01, ***p < 0.005, ****p < 0.001. (**D**) Myosin 3 (EmyHC) turnover per day (K) value during various stages of muscle regeneration. Significance determined by One-Way ANOVA with Benjamini–Hochberg correction for multiple comparisons. *p < 0.05, **p < 0.01, ***p < 0.005, ****p < 0.001.

To support our analysis of protein flux changes, we incorporated static abundance measurements of certain proteins that are only transiently expressed during muscle regeneration, along with our genome data, to confirm which stage of new myofiber genesis we are exploring. EmyHC protein expression was analyzed by immunofluorescence intensity on muscle sections that were taken at the end of each regeneration stage (Fig. 9A). The Proliferation Stage showed modest EmyHC expression, however, a significant increase in EmyHC expression was seen between the Proliferation Stage and the Differentiation Stage (Fig. 9B, p < 0.001). By the end of the Maturation Stage, the level of EmyHC expression was significantly lower than both the Proliferation Stage (Fig. 9B, p < 0.001) and the Differentiation Stage (Fig. 9B, p < 0.001), as most myofibers had undergone their initial formation stages and began to mature back into unaffected tissue. Protein levels of EmyHC were also measured from the fluorescent images and followed a similar pattern of significance as the abundance measurements expect for no significant difference between Proliferation and Differentiation Stages (Fig. 9B). Myh3, the gene for EmyHC, expression was also measured at the Proliferation Stage, and showed a significant increase in comparison to control values (Fig. 9C, p < 0.005). However, Myh3 gene expression no longer showed a significant difference from control levels at the Differentiation Stage and was significantly reduced from the Proliferation Stage (p < 0.005). These gene expression levels of Myh3 were also observed in the Maturation Stage, with levels that were like the control group and significantly lower than the Proliferation Stage (Fig. 9C, p < 0.001).

In addition, we investigated the relationship between kinetic measurements, protein abundance measurements, and gene expression values for this protein during the process of muscle regeneration. EmyHC FSR did not show a significant increase from control during the Proliferation Stage (Fig. 9D, p > 0.05), when protein abundance level measurements were only moderately increased (Fig. 9B) and Myh3 gene expression levels were significantly increased (Fig. 9C, p < 0.001). However, as with EmyHC abundance measurements, there was a significant increase in the EmyHC FSR value in the injured limb during the Differentiation Stage in comparison to the injured limb of the Proliferation Stage, as well as the control limb in the Differentiation Stage (Fig. 9D, p < 0.001). However, at this stage Myh3 gene expression was significantly decreased from the Proliferation Stage (Fig. 9C, p < 0.005). In the Maturation Stage, there was a significant decrease in EmyHC FSR from the injured limb of the Differentiation Stage to the injured limb of the Maturation Stage (p < 0.01). The same significant decrease between the two stages can be seen with both measurements of EmyHC protein abundance (number and staining intensity, Fig. 9B, p < 0.001). Myh3 gene expression was also significantly lower from previous stages (Proliferation Stage) and not significantly different from the control group. Interestingly, EmyHC from the injured limb during the Maturation Stage was still significantly higher in FSR values than control. This is different from what is seen in EmyHC protein abundance during the Maturation Stage, as it was the lowest of all the stages we measured. Also, there is no significant difference between the EmyHC FSR values of the injured limb from the Proliferation Stage and the injured limb from the Maturation Stage, which is different from what is seen in both EmyHC abundance and protein levels (Fig. 9B).

### TTN gene expression, titin protein turnover, and correlation between the two measurements

**Figure 10:**
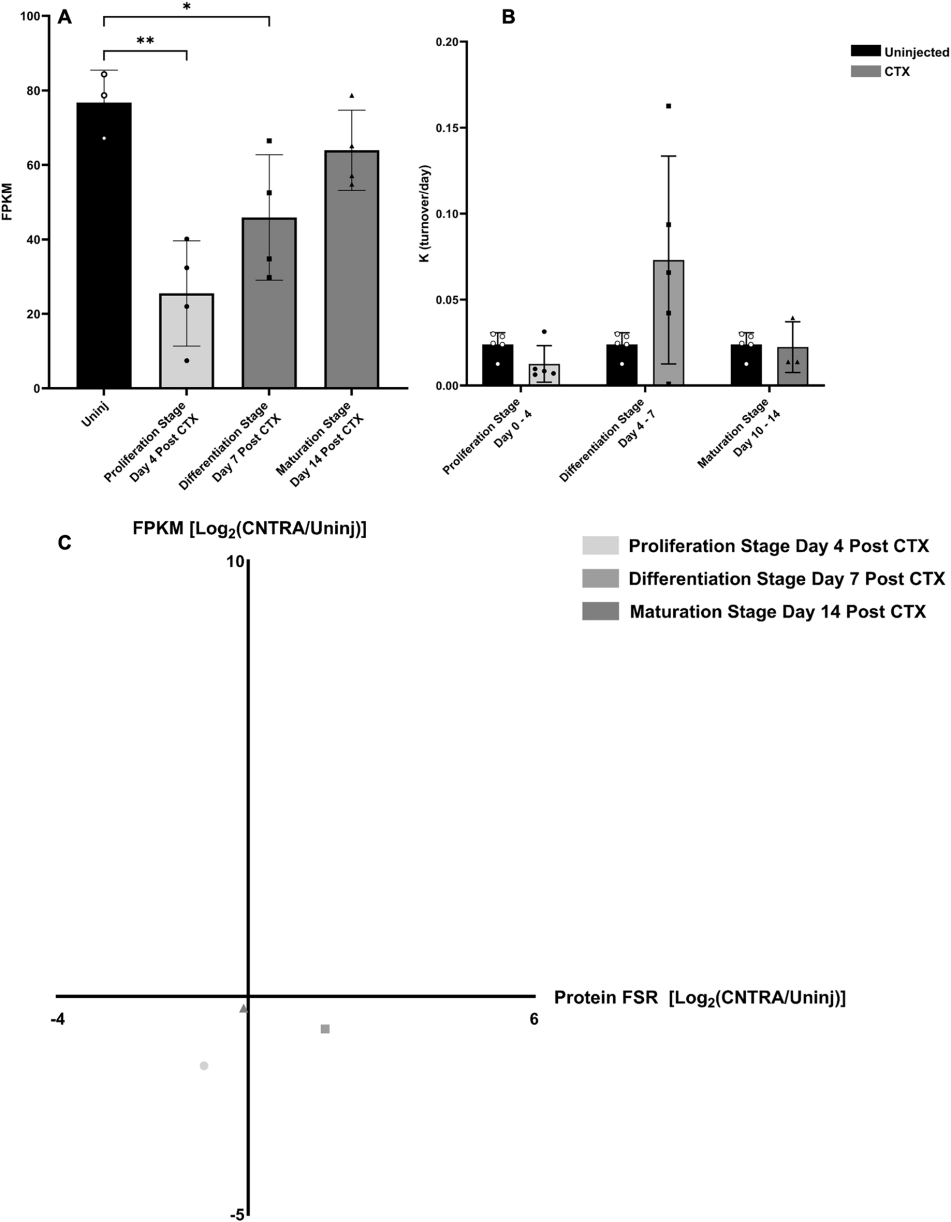
TTN gene expression and titin protein turnover. (**A**) Gene expression (FPKM) of TTN during various stages of muscle regeneration. Significance determined by One-Way ANOVA with Benjamini–Hochberg correction for multiple comparisons. *p < 0.05, **p < 0.01, ***p < 0.005, ****p < 0.001. (**B**) Titin protein turnover per day (K) value during various stages of muscle regeneration. Significance determined by One-Way ANOVA with Benjamini–Hochberg correction for multiple comparisons. *p < 0.05, **p < 0.01, ***p < 0.005, ****p < 0.001. (**C**) Correlation graph between log-transformed fold change in gene expression (FPKM, Y Axis) and log-transformed fold change in protein turnover (FSR, X Axis) for various stages of regeneration of the TTN gene to Titin protein.

To demonstrate the discordance between gene expression and protein turnover during muscle regeneration, we investigated the individual relationship of Titin (TTN) gene expression and protein turnover at various stages (Fig. 10A,B). Gene expression values of TTN show significant decreases at the Proliferation and Differentiation stages, with expression levels almost returning to normal by the Maturation Stage and being non-significant (Fig. 10A). However, this was not the case with Titin protein turnover, as there was no significant change between the individual protein’s turnover values at any stage of regeneration and the control groups (Fig. 10B). This general lack of correlation between gene expression and protein turnover is demonstrated well when the magnitude of change in both of these values are plotted together on a correlation graph (Fig. 10C).

## Discussion

Here, we describe how both protein flux changes and gene expression values are affected on global, group, and individual levels during sequential stages of muscle regeneration from CTX injury. This was achieved through paired “omics” approaches, including ^2^H_2_O labeling of proteins coupled to high-resolution MS analysis to measure in-vivo protein turnover rates and bulk RNA-sequencing of CTX injured tissue. To first validate our model of CTX-mediated initial muscle damage and subsequent regeneration, we used quantitative measurements of histology, DNA replication, and EmyHC expression profiles.

We utilized visual analysis of the injured tissue at each stage to confirm our model of induced injury caused sufficient necrotic damage and subsequent regeneration of that damaged muscle tissue. Previous work done by Mahdy et al. demonstrated histological analysis of CTX mediated muscle damage and regeneration on or around similar time points as our study^23^. The authors showed early, widespread tissue damage with fragmented myofibers and cellular edema that is followed by later-stage nascent myofiber formation and transient presence of central nuclei. Yan et al. also reported visual confirmation of new myofiber formation from MuSCs, with the abundance of central nuclei being the highest around 5–10 dpi^25^. In the later stages of muscle regeneration, the new myofibers have left their beginning stages of formation from MuSCs and have begun to mature into surrounding unaffected tissue. While this process generally begins after 7 dpi, we decided to delay the start of our measurement of protein fluxes until 10 dpi to allow for this process to develop. Visual analysis of the Maturation Stage shows significant decreases in measurements of histology and central nuclei, which indicates the early stage of myofiber genesis has ended. This point of muscle regeneration is where new tissue begins to develop the structures that surround the new myofiber to help integrate it back into the unaffected tissue^24^. By around 14 dpi, both papers report that newly formed myofibers are visibly indistinguishable from unaffected tissue, thus confirming the final stages of the regeneration process end around this time point.

To support our visual confirmation of unique stages of skeletal muscle damage and regeneration, ^2^H incorporation into DNA was measured from aliquots of single muscle fibers with attached fiber-associated cells to determine cell-cycle activity. Previous work shows that MuSCs cultured from mouse tissue ex-vivo that are responding to an injury model have high bromodeoxyuridine (BrdU) incorporation up to 3 dpi^27^, and that all cell populations actively incorporating BrdU at 3 dpi are MuSCs^25^. Our analysis shows high rates of cell-cycle activity from isolated muscle fibers based on high rates of DNA synthesis during the early stages of initial muscle damage (4dpi), but a striking and significant decrease in DNA synthesis rates as differentiation and muscle regeneration occurred. Interestingly, we only saw significant changes in the expression of the cell-cycling genes that we measured, including PCNA or Ki67, in the Differentiation Stage when PCNA had significant upregulation. However, gene expression of myogenic markers that are involved with newly formed myofibers, like MyoD and Myf5^8^, were high at early stages (Proliferation and Differentiation), but all decreased back to control levels by the Maturation Stage. Our results also support the findings from others^33^ that other myogenic genes of interest, such as Myozenin (Myoz1 and Myoz3), Troponin I (Tnni2), and Dystrophin (DMD), were significantly upregulated in their expression during the later stages of muscle regeneration (data not shown).

Interestingly, we did not see a significant difference in global protein flux rate changes during the Proliferation Stage, which encompasses the phases of initial cellular damage, the subsequent immune system response, and the activation/proliferation of MuSCs around damaged tissue. However, in later time points that capture the stages of novel myofiber genesis from the differentiation of MuSCs (Differentiation Stage) and reintegration of these new myofibers into existing tissue (Maturation Stage), we saw a very significant increase in global protein turnover rates at both stages in the CTX injured tissue compared to control. These results in global protein flux rate trends are a striking contrast to what we and others^25^ have observed with global gene expression analysis of damaged or regenerating muscle. The Proliferation Stage showed the highest total number of genes and percent of all genes discovered to be either significantly upregulated or downregulated. However, as the regeneration process occurred, the total number of genes and percent of all genes discovered being either significantly upregulated or downregulated decreased from Proliferation Stage to Differentiation Stage, and from Differentiation Stage to Maturation Stage. This trend of decreasing global gene expression as the muscle regeneration process occurs is in line with other works^25^ but is opposite of what we saw in protein turnover, which shows persistently high global changes into the later stages of muscle regeneration.

Along with global protein turnover measurements, proteins were grouped into functional clusters based on NIH DAVID ontology software to provide us with a deeper understanding of which specific cellular processes were most affected during each stage of the regeneration timeline. During the Proliferation Stage, only the proteins in the Myofibril functional cluster had a significant change in the direction of protein flux rates. This overall variance and lack of direction in group flux rates analysis may be due to the still persistent widespread tissue damage that is seen during this stage, which could influence turnover in specific protein groups^26^. In the Differentiation Stage, once the MuSCs began to differentiate towards new myofibers, there was a much more uniform effect seen with all measured functional clusters showing an across-the-board significant increase in protein turnover. At the Maturation Stage, group protein analysis showed significant increases in the number of proteins with higher turnover values for each functional cluster we measured. This is supported by the increase in global protein turnover rates at this stage as the myofiber still requires additional structure formation around it to integrate back into the unaffected tissue. This indicates that initial damage from CTX was still affecting the tissue, even at the later stages of regeneration while the tissue is maturing.

Gene expression values were also grouped into the same functional clusters as proteins based on matching genes to individual proteins in the groups. Interestingly, all the functional clusters that were measured for gene expression values in the Proliferation Stage were significantly decreased, except for Cytoplasm. Same with the Differentiation Stage, where we saw a different response in gene expression level than in protein turnover as all the functional clusters that were measured, besides Myofibril, had significantly more genes with a lower expression value compared to control. In the Maturation Stage, there were fewer groups than from the previous stages that we measured that have significant directional changes in gene expression and the overall magnitude of average change is less than the previous groups that we measured. This could be explained by the reduced global effect on gene expression at this stage as well. In summary, we found changes in group gene expression values that both fit previous findings from global abundance measurements at certain stages of regeneration but that did not correlate with actual protein synthesis rates.

On the individual protein level, we saw varying results in the number and types that were significant at each stage of regeneration. While there was not a significant change in global protein turnover values during the Proliferation Stage, a few individual proteins stand out as being significantly different. Our research group has previously identified proteins from skeletal muscle that are validated as a systemic biomarker of whole muscle FSR values (CA3)^28^. We then applied a similar approach to this work for identifying individual proteins of interest that had significantly different flux rates at one or multiple stages. Some of these proteins that had a significant change in FSR during the Proliferation Stage were involved in the access of cellular components to DNA to either promote or silence the DNA replication processes (Histone H2B Type 1-M)^29^, or individual glycolytic enzymes, like TPI. All data for other individual histone proteins can be found in Table 1. Along with significant increases in global protein flux rates at the Differentiation Stage, some individual proteins stood out as significant as well. After correction for multiple comparisons, 14 proteins stood out as individually significant. Proteins of interest that were significant at this stage include different Myosin Heavy Chain isoforms such as Myosin 3 and Myosin Light Chain 1/3. These might provide insight into the status of whole tissue response to injury at this stage^30–32^. Also, a handful of glycolytic enzymes (TPI, PGM-2, G3PDH, LDH-A) showed increases at this stage of regeneration, which have been previously found to be upregulated during the conversion of MuSC’s to myofibers^22^. Thus, suggests an increased demand for energy metabolism during this stage to support new myofiber formation, as further corroborated by functional cluster protein group changes mentioned above. In the later stages of a regenerative event, when nascent myofibers are tasked with maturing into surrounding tissue, we see a significant increase in both global protein flux rates and individual protein flux values. In the Maturation Stage, 91 individual proteins had a significantly different flux rate and all 91 proteins that were significantly different in the regenerating limb had a higher turnover value. Previously mentioned individual proteins of interest (CA3, CK-M, Myosin Light Chain 1/3) that could serve as non-invasive biomarkers had significantly increased protein turnover at this stage of regeneration as well. Interestingly, Myosin 3 protein fluxes were still high at this stage, after nascent myofibers began to mature^31^. Also, Histone H2B Type 1-M is seen to have a significant increase in this stage as well. Recent literature suggests that ribosomal biogenesis is important for muscle tissue hypertrophy, such as what is seen in tissue regeneration from damage^34^. However, we were not able to measure any ribosomal proteins in any of the stages that we analyzed during tissue regeneration. Therefore, we cannot support these previous findings in this article. From this analysis of individual protein flux changes along an in-vivo muscle regeneration time course, we were able to discover more information about what is occurring at each stage along this temporally regulated process. Each one of these individual proteins that we have identified here as having a significant change in its flux rate at one or multiple stages is of relevant interest because they can serve as a potential biomarker of whole tissue FSR values, a surrogate measurement for the cellular process it is a part of, or a possible target for therapeutic modulation at that stage of muscle regeneration.

Knowing what individual protein turnover values stood out as significant after correction for multiple comparisons, we explored the relationship between protein turnover values and their gene expression values at the end of each labeling period. A correlation graph that compares the magnitude of change in an individual protein’s turnover value and the magnitude of change in gene expression values was made for each stage. For the Proliferation Stage, 71 proteins had available turnover and gene expression data but there was no significant correlation between the two types of measurements. In the Differentiation Group, for 124 proteins that had available turnover and gene expression data, there was no significant correlation between the two types of measurements. In the Maturation Stage, 121 proteins had available turnover and gene expression data, and there was no significant correlation between the two types of measurements. Across all stages that we measured during muscle regeneration, there was no significant correlation between the magnitude of change in protein turnover values and gene expression values. This divergence in change between protein turnover and gene expression may be explained by post-transcriptional levels of control over protein synthesis (e.g., initiation or elongation translational changes, microRNA effects, etc.) or by delays in gene transcript turnover, whereas protein synthesis rates measure the current flux rate of each protein. This evidence of translational control during muscle regeneration is an important finding^35–37^. In fact, a few recent studies from our research group showed a clear dissociation between protein turnover and gene expression levels in various tissues including skeletal muscle and liver^38,39^.

Specific time-dependent protein abundances, along with gene expression profiles, were also measured to substantiate our findings at each measured stage of the regeneration process. As mentioned previously, EmyHC is a protein that is transiently expressed from the gene Myh3 during the nascent stages of new myofiber formation, after proliferating MuSCs begin to differentiate^31^. Previous findings^40^ support our results of a moderate abundance of EmyHC protein at 4 dpi, but a significantly higher protein abundance measurement at 7 dpi. Interestingly, fluorescent-based protein expression levels from individual myofibers don’t show a significant increase in values between these two stages. These protein abundance and level values of EmyHC started to decrease after about 7 dpi, as new myofibers began to mature into existing tissue^31^ and return to almost baseline by the end of our regeneration time course at 14 dpi. EmyHC protein abundance patterns have also been found to mirror that of central nuclei abundance in the earlier stages of regeneration^41^. We saw a similar correlation in expression between markers of new myofibers, EmyHC and central nuclei, which decreased from their high co-abundance at the Differentiation Stage, almost back to baseline levels by the Maturation Stage. However, protein abundances lag a bit behind gene expression values as there was a highly significant increase in Myh3 gene expression, which started to decrease after 4 dpi and is no longer significant from control by 7 dpi or 14 dpi. Unlike EmyHC, we did not see any form of correlation between gene expression and protein turnover with another muscle-specific protein Titin. The relationship between the two values for this protein further demonstrates the overall lack of correlation that we found between gene expression and protein turnover at all stages of regeneration.

Deeper analysis of the differentially expressed genes (either upregulation or downregulation) at each stage was done to understand which biological processes were impacted significantly by an acute muscle tissue injury. In the Proliferation Stage, both lists with downregulated and upregulated genes have significant group enrichment. Some of the GOBP’s that were downregulated and significantly enriched include catabolism and negative regulation of cellular processes. Some of the GOBP’s that were upregulated and significantly enriched include immune response and inflammatory processes. This would make sense in the context of this stage as there would still be recovery from initial tissue damage. This stage also encompassed the activation and proliferation of MuSCs around the still injured tissue. Perhaps the cellular proliferation processes have ended by Day 4, when these gene enrichment analyses were done, and such represents a decrease in these processes. In the Differentiation Stage, both the upregulated and downregulated differentially expressed genes list have significant group enrichment. GOBP’s that were differentially downregulated and significantly enriched were metabolic and catabolic processes. GOBP’s that were differentially upregulated and significantly enriched were intracellular and extracellular structure formation. This makes sense as the old, damaged tissue is being removed and new tissue is starting to form in its place from MuSCs at this stage of muscle regeneration. In the Maturation Stage, both the upregulated and downregulated differentially expressed genes list have significant group enrichment. GOBP’s that were differentially downregulated and significantly enriched were catabolic processes and DNA replication. GOBP’s that are differentially upregulated and significantly enriched were intracellular and extracellular structure formation. In the context of the Maturation Stage, these significantly enriched groups make sense because this is when newly differentiated tissues mature by forming the surrounding structures necessary to incorporate back into undamaged tissue.

## Conclusions

Here, we present a new method of using stable isotope labeling and tandem mass spectrometry to measure changes in protein turnover rates during sequential and unique stages of in-vivo muscle regeneration. Global, ontology-grouped, and individual turnover rates from tissue were then compared directly to gene expression values from mRNA sequencing to examine their relationship throughout the muscle regeneration process. The results here provide a novel characterization of protein turnover rates, and their comparison to gene expression values, at different levels (global, grouped, and individual) and different stages of muscle regeneration. This research deepens the available knowledge of the in-vivo muscle regeneration process, which should help advance progress in related fields, such as anti-aging/longevity or muscular disorders.

## Limitations

There are various limitations of this study that the authors acknowledge might produce shortcomings to the overall outcomes of the work. First, the study exclusively used male mice for the research. There are various physiological differences between male and female mice, including with muscle tissue regenerative capacity, which could influence the results of this study for different genders. Also, while the amount and technique used to inject CTX was standardized across all subjects, it is difficult to damage exactly the same amount of tissue with each injection. Proper precautions were taken to mitigate the impact of all of the recognized study limitations as much as possible.

## Data Availability

The datasets generated and/or analyzed during the current study are available in the following online repositories: RNA-seq: NCBI GEO, Accession: GSE261089, https://www.ncbi.nlm.nih.gov/geo/query/acc.cgi?acc=GSE261089. Proteomics: jPOST, Accession: JPST002986^42^, https://repository.jpostdb.org/entry/JPST002986. Supplemental Materials can be found here: 10.6084/m9.figshare.25356613.v1 ^43^.

## Abbreviations

^2^H_2_O: Deuterated water
MS: Mass spectrometry
CTX: Cardiotoxin
mRNA-seq: Next generation mRNA-sequencing
MuSCs: Muscle satellite cells
HPLC: High-performance liquid chromatography
MS/MS: Tandem mass spectrometry
MIDA: Mass Isotopomer Distribution Analysis
ACUC: Animal Care and Use Committee
TA: Tibialis anterior
OCT: Optimal cutting temperature
PBS-T: Phosphate buffered solution with trition X-100
NDS: Normal donkey serum
BSA: Bovine serum albumin
GC-MS: Gas chromatography mass spectrometry
QC: Quality control
MACS: Magnetic assisted cell sorting
TCEP: Tris-(2-carboxyethyl)-phosphine
IAA: Iodoacetamide
LC-MS: Liquid chromatography–mass spectrometry
ESI: Electron spray ionizing source
Q-ToF: Quadropole time of flight
FSR: Fractional synthesis rate
DPI: Days post-CTX injection
H&E: Hematoxylin & eosin
SD: Standard deviation
EmyHC: Embryonic myosin heavy chain
PGM-2: Phosphoglycerate mutase 2
G3PDH: Glyceraldehyde-3-dehydrogenase
LDH-A: Lactate dehydrogenase A
CA3: Carbonic anhydrase 3
TPI: Triose phosphate isomerase
CK-M: Creatine kinase-muscle isoform
FPKM: Fragment per kilobase millions
GOBP: Gene ontology biological processes
Log_2_FC: Log_2_(fold change)
Tnni2: Troponin I
DMD: Dystrophin
BrdU: Bromodeoxyuridine
TTN: Titin
